# Identifying the programmed cell death index of hepatocellular carcinoma for prognosis and therapy response improvement by machine learning: a bioinformatics analysis and experimental validation

**DOI:** 10.3389/fimmu.2023.1298290

**Published:** 2023-12-19

**Authors:** Yuanxin Shi, Yunxiang Feng, Peng Qiu, Kai Zhao, Xiangyu Li, Zhengdong Deng, Jianming Wang

**Affiliations:** ^1^ Department of Biliary and Pancreatic Surgery/Cancer Research Center Affiliated Tongji Hospital, Tongji Medical College, Huazhong University of Science and Technology, Wuhan, China; ^2^ Department of Pediatric Surgery, Tongji Hospital of Tongji Medical College, Huazhong University of Science and Technology, Wuhan, China; ^3^ Affiliated Tianyou Hospital, Wuhan University of Science and Technology, Wuhan, China

**Keywords:** hepatocellular carcinoma, immunotherapy, prognostic model, machine learning, programmed cell death

## Abstract

**Background:**

Despite advancements in hepatocellular carcinoma (HCC) treatments, the prognosis for patients remains suboptimal. Cumulative evidence suggests that programmed cell death (PCD) exerts crucial functions in HCC. PCD-related genes are potential predictors for prognosis and therapeutic responses.

**Methods:**

A systematic analysis of 14 PCD modes was conducted to determine the correlation between PCD and HCC. A novel machine learning-based integrative framework was utilized to construct the PCD Index (PCDI) for prognosis and therapeutic response prediction. A comprehensive analysis of PCDI genes was performed, leveraging data including single-cell sequencing and proteomics. GBA was selected, and its functions were investigated in HCC cell lines by *in vitro* experiments.

**Results:**

Two PCD clusters with different clinical and biological characteristics were identified in HCC. With the computational framework, the PCDI was constructed, demonstrating superior prognostic predictive efficacy and surpassing previously published prognostic models. An efficient clinical nomogram based on PCDI and clinicopathological factors was then developed. PCDI was intimately associated with immunological attributes, and PCDI could efficaciously predict immunotherapy response. Additionally, the PCDI could predict the chemotherapy sensitivity of HCC patients. A multilevel panorama of PCDI genes confirmed its stability and credibility. Finally, the knockdown of GBA could suppress both the proliferative and invasive capacities of HCC cells.

**Conclusion:**

This study systematically elucidated the association between PCD and HCC. A robust PCDI was constructed for prognosis and therapy response prediction, which would facilitate clinical management and personalized therapy for HCC.

## Introduction

Hepatocellular carcinoma (HCC) continues to be a leading cause of cancer-associated mortality, with its incidence increasing annually at a rapid rate. It is projected that by 2025, nearly one million new cases will be reported (1, 2). Standardized treatments such as surgical resection or liver transplantation for early-stage tumors, transarterial chemoembolization (TACE) for intermediate-stage tumors, and systemic therapies, including tyrosine kinase inhibitors (TKIs) and immune checkpoint inhibitors (ICIs) for advanced-stage tumors (3), have enhanced the prognosis of patients with HCC. However, the outcomes are still often short of expectations. Historically, clinical staging systems, such as the Barcelona Clinic Liver Cancer (BCLC) staging system, have played a central role in HCC management, serving as routine tools for clinicians to evaluate the conditions and therapeutic requirements of patients in practice (4). Nevertheless, the current clinical staging systems have limitations that may hinder their capacity to provide optimal therapeutic interventions to patients. They only focus on clinicopathological characteristics and do not take into account an individual’s molecular biological characteristics (5). Therapeutic decisions relying solely on them were obviously unilateral and could lead to potential over- or undertreatment, contributing to suboptimal therapeutic outcomes. For HCC, which is characterized by high heterogeneity, the realization of personalized treatment is essential to improving patient prognosis (6, 7). Thus, it is imperative to identify novel biomarkers that can clarify the molecular biological profile of patients, aid in risk stratification, and ultimately optimize HCC treatments and prognosis.

Programmed cell death (PCD), also referred to as regulated cell death, is the gene-regulated autonomous process employed by cells to maintain homeostatic balance. The progression and treatment response of tumors are intricately associated with PCD. Broad crosstalk exists in the initiation and regulation of various PCD types, and this interaction has emerged as a prominent focus in tumor research. Alongside the recently identified disulfidptosis and cuproptosis, the mainly recognized types of PCD include apoptosis, necroptosis, ferroptosis, pyroptosis, autophagy, parthanatos, entosis, NETosis, lysosome-dependent cell death, alkaliptosis, and oxeiptosis (8). Disulfidptosis was discovered in UMRC6 cells characterized by high *SCL7A11* expression. It occurs under conditions of glucose deficiency, resulting in the accumulation of disulfide bonds, which cause abnormal cross-linking between actin and cytoskeletal proteins. Consequently, this leads to cytoskeletal contraction and the collapse of the actin network, ultimately resulting in cell death (9). Cuproptosis is induced by an overload of copper ions, and its regulation is closely tied to mitochondrial metabolism and the sulfuric acid pathway (10, 11). Apoptosis is the most classical form of PCD and is the primary target of current antitumor strategies (12). Anoikis is a specific case of intrinsic apoptosis, triggered by the loss of cellular contact with the extracellular matrix or other adjacent cells. It serves as an important inhibitor in the growth and metastasis of tumors (13–15). Necroptosis is considered an alternative mechanism to apoptosis, primarily mediated by *RIPK1*, *RIPK3*, and *MLKL*, and can be inhibited by Nec-1. Necroptosis plays a dual role in tumors, as it can inhibit tumor growth while promoting metastasis and immune suppression through inflammatory responses induced (16, 17). Ferroptosis is a cell death type resulting from iron-dependent lipid peroxidation. Targeting ferroptosis represents a promising antitumor strategy (18, 19). Pyroptosis is mediated by the *gasdermin* protein family and is also associated with tumor proliferation and metastasis (20). The potential anti-tumor effects of pyroptosis have gained increasing attention (21). The occurrence of autophagy relies on lysosomal degradation, and its role in tumors is complex (22). On the one hand, autophagy is an important mechanism for suppressing tumor formation, but once a tumor is established, the activation of autophagy could promote further progression (23). Parthanatos is a cell death reliant on PARP-1 and is widely implicated in pathological processes such as inflammatory damages and neoplasms leading to aberrant activation of PARP-1 (24). Entosis, initially discovered in certain tumors, is described as a phenomenon of cell cannibalism (25). NETosis is a specialized mechanism in neutrophils for resisting pathogens, characterized by the formation of neutrophil extracellular traps (NETs) through the release of chromatin covered with antibacterial proteins (26, 27). Lysosome-dependent cell death is often induced by an imbalance in the cellular internal environment, marked by lysosomal membrane permeabilization and the release of lysosomal contents into cytoplasm (28). Alkaliptosis was discovered during antitumor molecular screening of G protein-coupled receptors, and it is regulated by an elevation of intracellular pH levels (29, 30). Oxytosis is cell death mediated by reactive oxygen species, with *KEAP1-PGAM5-AIFM1* as the key axis regulating this process (31).

Owing to its close association with tumors, PCD has become a central focus in the field of oncology research. However, comprehensive studies elucidating the relationship between PCD and HCC remain lacking. In the study, we performed a summative analysis of 14 PCD modes within HCC and developed the programmed cell death index (PCDI) using a machine learning algorithms-integrated framework. The PCDI could effectively characterize the heterogeneity of HCC patients, enabling risk stratifications among them and accurate prediction of their clinical prognosis and therapeutic response. This, in turn, could facilitate the personalized treatment and clinical management for HCC.

## Materials and methods

### Data collection and processing

The regulatory factors that govern 14 PCD modes were identified as PCD-related genes (**Supplementary Table S1**). These genes were sourced from the GSEA gene sets, KEGG, previous studies (32), and the Gene-Cards online platform (https://www.genecards.org/). A total of 1,937 nonredundant PCD-related genes were included for analysis.

Three independent HCC datasets containing clinical and transcriptomic data of patients, TCGA-LIHC, GSE76427, and ICGC-LIRI-JP, were acquired from TCGA database (https://portal.gdc.cancer.gov/), GEO database (https://www.ncbi.nlm.nih.gov/geo/), and ICGC database (https://icgc.org/), respectively. The transcriptomic data underwent conversion into TPM values using the “limma” package, followed by the removal of batch effects using the “SVA” package. Subsequently, the log2 transformation was conducted. A total of 711 HCC samples were included for analysis: 365 from TCGA-LIHC dataset, 231 from the ICGC-LIRI-JP dataset, and 115 from the GSE76427 dataset (**Supplementary Table S2**). TCGA-LIHC dataset served as the training dataset, while the GSE76427 and ICGC-LIRI-JP datasets were employed as validation datasets for the construction and evaluation of the PCDI.

### Analysis of expression patterns and mutation characteristics of PCD-related genes

The “limma” package was employed to identify differentially expressed genes (DEGs) with these criteria of *p* < 0.05 and |log2FC| > 1. A univariate Cox analysis was performed to identify prognostic genes, which were used in subsequent analyses. Mutation characteristics of prognostic PCD genes were described using the “mafTools” package. The copy number variation (CNV) characteristics of these genes were visualized through the GISTIC algorithm and the “RCircos” package.

### Identification of PCD clusters

Unsupervised clustering analysis was performed to identify the distinct PCD clusters in HCC patients. PCA, t-SNE, and UMAP analyses were utilized to illustrate the differences in sample distribution between PCD clusters. The survival analysis was performed using the R packages “survival” and “survminer”. The “Pheatment” package was utilized to visualize the expression patterns of PCD-related genes, immune checkpoint genes (ICGs), chemotherapy resistance-related genes (CRRGs), and clinicopathological characteristics between different PCD clusters. ICGs and CRRGs were obtained from previous studies (33, 34) and the Gene-Cards website. The “ESTIMATE” package was applied for calculating the TME score of patients, and their immune cell infiltration levels were evaluated through the ssGSEA algorithm.

### Functional enrichment analysis

We employed various methods to elucidate the biological functional differences among HCC patients. For HCC patients in different PCD clusters, Gene Set Variation Analysis (GSVA), Gene Set Enrichment Analysis (GSEA), and GO/KEGG functional enrichment analyses were all used. The same methods were employed in the analysis of patients with different PCDI scores. GSEA was also applied to explore the potential functions of PCDI genes in patients with HCC. The criteria for GSVA and GO/KEGG analyses were both *p*-value < 0.05 and FDR < 0.05; for GSEA, the criteria were *p*-value < 0.05, FDR < 0.25, and NES > 1.

### Construction and prognostic predictive value evaluation of the PCDI

To develop an accurate and robust PCDI, the following steps were adopted:

1. Using the univariate Cox analysis, 87 prognostic PCD genes were introduced for prognostic model construction.
2. We employed a machine learning algorithm integrated framework that incorporated 10 machine learning algorithms, such as random survival forest (RSF), partial least squares regression for Cox (plsRcox), supervised principal component (SuperPC), generalized boosted regression modeling (GBM), support vector machine (SVM), elastic net (Enet), LASSO, ridge, stepwise Cox, and CoxBoost. Via 10-fold cross-validation, we generated 88 algorithm combinations within TCGA-LIHC dataset for training prognostic models, and further validation was carried out in the GSE76427 and ICGC-LIRI-JP datasets. Upon comparison, the model that exhibited the highest average C-index among these three datasets was thus determined as the PCDI.
3. In this study, the PCDI was constructed through the combination of CoxBoost and RSF algorithms. The CoxBoost model was instantiated utilizing the “CoxBoost” software package, engineered to facilitate the estimation of Cox proportional hazards models through componentwise likelihood-based boosting techniques. For this model, the optimal regularization parameter, signifying the extent of shrinkage, was rigorously identified by employing the 10-fold cross-validation strategy within the framework of the CoxBoost penalty function. The “RandomForestSRC” package was employed for the RSF model. This model comprised two parameters. Ntree was indicative of the number of trees constituting the forest, and mtry represented the quantity of arbitrarily selected variables designated for bifurcation at every individual node. A meticulous grid search was conducted on both ntree and mtry, assisted by the 10-fold cross-validation mechanism. All possible pairings of (ntree, mtry) were formulated, with the pairing boasting the superior C-index value recognized as the optimized parameters.
4. A comprehensive evaluation was subsequently carried out to assess the prognostic value of PCDI. Patients were categorized into dichotomous groups based on their PCDI score. Survival curves were generated to compare the prognosis between the two groups. ROC curves were applied to assess the predictive accuracy of PCDI, while chi-square analysis was performed to explore the correlation between PCDI and other clinicopathological features. The independent prognostic value of PCDI and other clinicopathological factors was compared through univariate and multivariate Cox analyses. The predictive efficacy of PCDI and other clinicopathological attributes was assessed through C-index curves and DCA curves. Additionally, the predictive efficacy of PCDI was compared with 102 other published prognostic models using C-index curves (**Supplementary Table S12**).

### Construction and evaluation of the clinical nomogram

The “rms” and “regploy” packages were used to develop a clinical nomogram based on the PCDI and other clinicopathological factors, predicting the overall survival (OS) of patients with HCC. Calibration and ROC curves along with DCA were used to evaluate the predictive efficacy of the clinical nomogram.

### Correlation analysis of PCDI with immunological, gene mutation, and stemness characteristics

Using various algorithms, including CIBERSORT-ABS, TIMER, and XCELL, we assessed the differences in immune cell infiltration levels between these two groups. The “ESTIMATE” package was utilized to calculate the tumor microenvironment (TME) score, while Gene Set Variation Analysis (GSVA) and single sample gene set enrichment analysis (ssGSEA) were performed to further explore the immunological functional status. Additionally, the correlation between PCDI and ICG expression patterns was investigated.

The “maftools” package was applied to describe different mutation statuses of patients between both groups. We also compared their different TMB and microsatellite instability (MSI) statuses. Moreover, we extracted the stemness index of HCC patients from “StemnessScores_RNAexp_20170127.2.tsv”. Subsequent correlation analysis was performed between the PCDI and tumor stemness features.

### Predictive value evaluation of the PCDI in immunotherapeutic responses

Employing the Tumor Immune Dysfunction and Exclusion (TIDE) algorithm (http://tide.dfci.harvard.edu/) and multiple immunotherapy cohorts, we discussed the value of the PCDI in immunotherapeutic response prediction. In TCGA-LIHC dataset, we calculated and compared the TIDE, dysfunction, and exclusion scores of HCC patients in the two groups. A correlation analysis between immunotherapeutic response and PCDI score was then performed. Subsequently, the predictive capability of PCDI for immunotherapeutic response was further validated in 10 cohorts: IMvigor210 (35), Checkmate (36), GSE175307, GSE179351, GSE165252, GSE103668, GSE78220, GSE91061, GSE35640, and GSE120644, which included the immunotherapeutic response data from tumor patients. Moreover, the GSE109221 cohort (sorafenib treatment for HCC) and GSE104580 cohort (TACE treatment for HCC) were included for an extensive assessment of the predictive value in HCC treatments.

### Correlation analysis between PCDI and chemotherapeutic drug sensitivities

In TCGA-LIHC dataset, we detected the different expression patterns of CRRGs between patients in the high and low PCDI score groups. Furthermore, the “OncoPredict” package was applied in predicting various chemotherapeutic drug sensitivities between the two groups.

### PCDI gene analysis based on single-cell transcriptomic data

GSE125449 was obtained from the GEO database, which encompassed single-cell transcriptomic profiles from 19 liver cancer patients. The “Seurat” package was employed for the initial data processing. For the GSE125449 dataset, quality control was conducted according to these criteria: (1) genes expressing in fewer than three cells were excluded; (2) cells expressing fewer than 500 genes were excluded; (3) cells expressing 500 to 10,000 genes were retained; (4) cells with mitochondrial gene expression exceeding 20% were excluded; and (5) cells with ribosomal gene expression exceeding 20% were excluded. The “NormalizeData” function was applied to normalize the data passed quality control measures. Highly variable genes were identified by the “FindVariableFeatures” function. The “ScaleData” function was utilized for scaling gene expression profiles. Dimensionality reduction was executed using the “RunPCA” function, and the first 20 principal components (PCA) were selected for cluster analysis. The main cell types were annotated utilizing the “SingleR” package, with subsequent corrections based on markers in the original literature (37). The “CellChat” package was employed to assess cellular communication among different cell populations.

### PCDI gene analysis based on proteomic and immunohistochemistry data

The HCC proteomic dataset PDC-000198 was obtained from the CPTAC database (https://pdc.cancer.gov/pdc/), with 151 samples with complete clinical information and proteomic data included. Using the “limma” package and the criteria of *p* < 0.05 and |log2FC| > 0.585, we assessed the different expression patterns of PCDI genes between tumor and adjacent tissues at the protein level. Survival analysis was performed as described before. And immunohistochemistry data of PCDI genes was acquired from the Human Protein Atlas(HPA) database (https://www.proteinatlas.org/) for further analysis.

### Cellular cultivation and transfection

The human HCC cell lines MHCC97H and HuH-7 were acquired from the Hepatic Surgery Center at the Affiliated Tongji Hospital of Huazhong University of Science and Technology. All cells underwent rigorous STR analysis to ensure they were free from mycoplasma contamination. HCC cells were cultured with Dulbecco’s modified Eagle’s medium (DMEM) (Cibco, Massachusetts, USA) added the 10% fetal bovine serum (FBS) (Gibco, USA) under the conditions of 37°C and 5% CO_2_ atmospheric composition.

SiRNAs were transfected into MHCC97H and HuH-7 cells to downregulate *GBA* expression. The negative control siRNA (si-NC), si-*GBA*-1, si-*GBA*-2, and si-*GBA*-3 were designed and synthesized by Hippo Biotechnology (Huzhou, China), with detailed sequences provided in **Supplementary Table S3**. HCC cells under optimal conditions were seeded uniformly into six-well plates. Upon cell adhesion and achieving approximately 50% confluency, transfection was executed utilizing Lipofectamine 2000 (Invitrogen, Massachusetts, USA).

### HCC tissue sample collection

Five paired HCC tumors and adjacent tissue samples were obtained from the Affiliated Tongji Hospital of Huazhong University of Science and Technology with the ethical authorization conferred by the Tongji Hospital Research Ethics Committee. The information on HCC patients is delineated in **Supplementary Table S4**.

### Quantitative real-time PCR and Western blotting

The total RNA extraction was conducted with the TRIzol reagent (Vazyme, Nanjing, China). CDNA synthesis was carried out with ABScript III RT Master Mix (ABclonal, Wuhan, China). Quantitative real-time PCR (qRT-PCR) analysis was performed with Universal SYBR Green Fast qPCR Mix (ABclonal) in the CFX96 Touch™ Real-Time PCR Detection 203 System (Bio-Rad, California, USA). GAPDH served as the internal negative control, and the relative mRNA expression levels of target genes were quantified with the 2^−ΔΔCT^ method.

Western blotting (WB) was carried out following the published protocols previously (38), and Image Lab software (Bio-Rad, California, USA) was used in data analysis. GAPDH served as the internal negative control for the comparison of protein expression levels across various groups. The primers and antibodies involved in this study are listed in **Supplementary Table S5**.

### Functional experiments on proliferation, invasion, and migration *in vitro*

Cell Counting Kit-8 (CCK-8, ABclonal, Wuhan, China) assay and colony formation test were utilized for assessing the proliferative capacity of HCC cells. For the CCK-8 assay, MHCC97H and HuH-7 cells were seeded in 96-well plates at 3,000 cells/well density. Upon cell adhesion, the medium was substituted with DMEM supplemented with CCK-8 reagent (100 µL DMEM + 10 µL CCK-8 per well). Each group had five duplicate wells. The absorbance at 450 nm was measured after a 2-h incubation at 37°C. The CCK-8 assay spanned 3 days. For the colony formation test, HCC cells were seeded in six-well plates at 500 cells/well density. The culture medium was replaced every 3 days, following the same cell cultivation procedure as previously described. Cultivation was terminated after 2 weeks, and the cell colonies were fixed with paraformaldehyde (Solarbio Science and Technology Co., Beijing, China) for 25 min, followed by staining with the crystal violet dye (G1014, Servicebio, Wuhan, China) for 25 min. Cell colonies were counted under a microscope.

The Transwell assay and wound-healing test were both used for assessing the migratory and invasive capacity of HCC cells. For the transwell assay, MHCC97H and HuH-7 cells were cultured in a serum-free medium for 8 h. Subsequently, 5*10^4^ cells were resuspended in 200 µL of serum-free medium and uniformly seeded to the upper chamber of Transwell inserts (Corning, New York, USA), with Matrigel coating (BD Bioscience, New Jersey, USA) for invasion or with no Matrigel coating for migration. The lower chamber was filled with 700 µL of complete DMEM medium. After culturing for 36 h, the chambers were harvested. Cells that invaded or migrated to the lower surface of the chamber were fixed and stained as previously described. Cellular migration or invasion was quantified with ImageJ software, with the calculation of average cell counts from five randomly selected fields of view.

For the wound-healing test, HCC cells were uniformly seeded in six-well plates. Upon reaching a cellular confluence exceeding 95%, scratches were performed with a 200-µL pipette tip. At 0 h, 12 h, 24 h, and 48 h, nonadherent cells were removed carefully, and photographs were captured. The scratch closure rates were analyzed with ImageJ software.

All experiments were independently replicated three times.

### Statistical analysis

In this study, statistical analysis was accomplished with R 4.3.0 and GraphPad Prism 8.0.1 software. The findings of *in vitro* experiments were typified by representative images from three independent replicates, conveyed as the mean ± standard deviation (SD). The Spearman’s correlation coefficient was conducted for the correlation test between continuous variables. The Chi-square test was utilized to assess the correlation between categorical variables. The differences between groups were determined by the Wilcoxon rank-sum test, independent Student’s *t*-test, or analysis of variance for continuous variables. The survival analysis was performed employing the Kaplan–Meier (KM) method, and the log-rank test was applied for the assessment of statistical significance. A *p*-value of < 0.05 indicated statistical significance.

## Results

### Landscape of expression and mutation in PCD-related genes

The comprehensive framework for this present study is depicted in **Supplementary Figure S1**.

**Figure 1:**
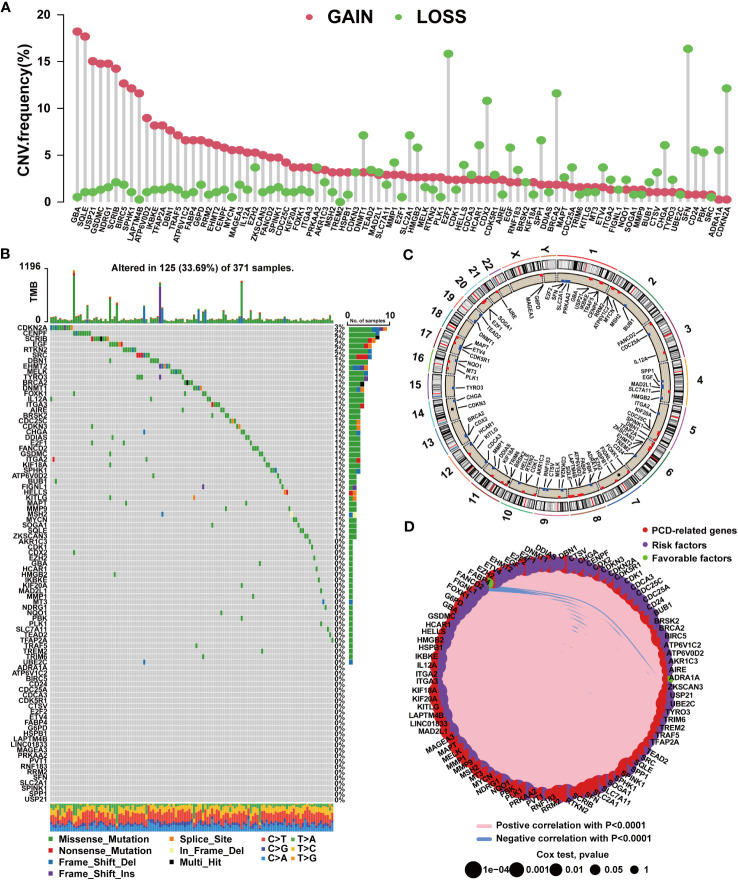
Mutation characteristics of PCD-related genes in HCC. **(A)** Characteristics of CNVs in PCD-related genes. **(B)** Characteristics of genetic variation in PCD-related genes. **(C)** Location of CNVs in PCD-related genes on chromosomes. **(D)** Expression correlation among PCD-related genes.

By analyzing PCD-related gene expression profiles, we identified 756 DEGs. Among these, 721 genes exhibited upregulated expression in tumor tissues, while only 35 genes displayed downregulated expression (**Supplementary Table S6**). We further conducted univariate Cox regression analysis, revealing 87 prognostic PCD-related genes. Among these, 85 genes correlated with an unfavorable prognosis in HCC, while *ADRA1A* and *FABP4* were protective factors for patients (**Supplementary Table S7**). A subsequent analysis of the 87 prognostic PCD genes was conducted. As shown in **Figure 1A**, these PCD-related genes frequently exhibit CNVs. The top 5 genes with the highest amplification frequencies were *GBA*, *SQLE*, *USP21*, *GSDMC*, and *NDRG1*, while *SFN*, *E2F2*, *CDKN2A*, *BRCA2*, and *CDX2* displayed the highest frequencies of copy number loss. The chromosomal locations of CNVs are presented in **Figure 1C**. Additionally, we observed that PCD-related genes exhibited mutations in 125 samples, with *CDKN2A* exhibiting the highest mutation frequency (**Figure 1B**). **Figure 1D** depicts the expression network of the aforementioned PCD-related genes.

### Identifying PCD clusters with distinct characteristics of clinicopathology, molecular patterns, and functions

**Figure 2:**
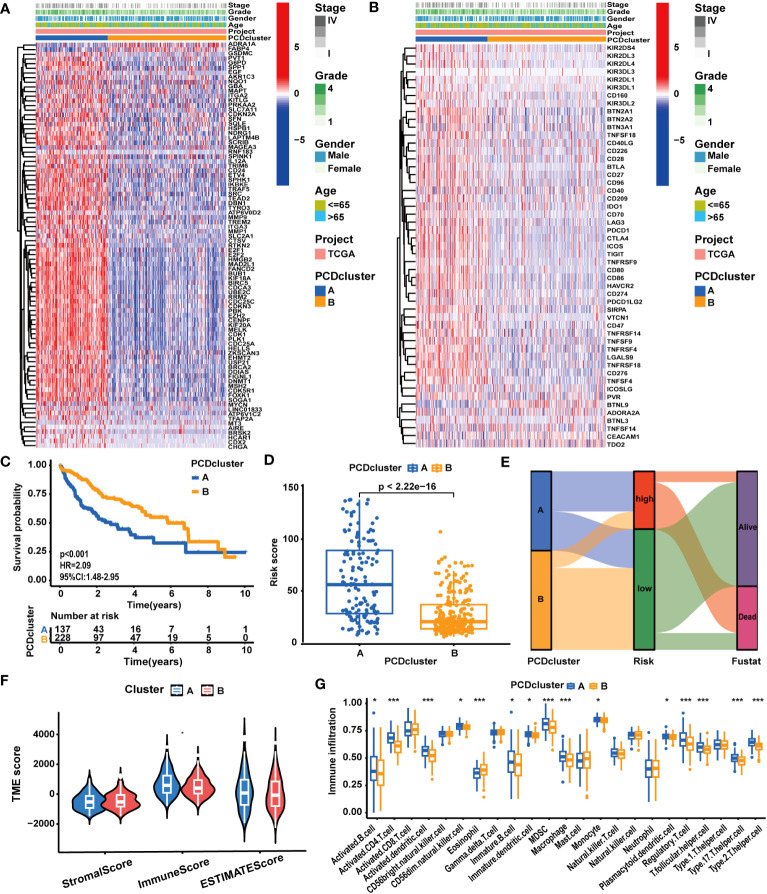
The correlation between PCD clusters, clinicopathological characteristics, and molecular patterns. **(A)** Different clinicopathological characteristics and PCD-related gene expression patterns between the two PCD clusters. **(B)** Different clinicopathological characteristics and ICG expression patterns between the two PCD clusters. **(C)** Different OS statuses of HCC patients between the two PCD clusters. **(D)** Correlation analysis between PCDI scores and PCD clusters. **(E)** Distribution of patients with different OS statuses across PCD clusters and PCDI score groups. **(F)** Different TME scores between the two PCD clusters. **(G)** Different immune cell infiltration patterns between the two PCD clusters. (^*^ *p* < 0.05; ^**^ *p* < 0.01; ^***^ *p* < 0.001.).

In accordance with the expression profiles of the 87 prognostic PCD genes, we identified two PCD clusters (**Supplementary Figures S2A–D**). PCA, t-SNE, and UMAP analysis substantiated notable disparities in the distribution of patient samples between the two PCD clusters (**Supplementary Figures S2E–G**). As illustrated in **Supplementary Figure S3A** and **Figure 2A**, we found patients in cluster A exhibited higher expression levels of PCD-related genes and suffered advanced clinical stages and pathological grades. In **Figure 2C**, survival curves clearly demonstrate that patients in cluster A experienced worse survival outcomes. Concurrently, it was demonstrated that the expression levels of most ICGs and CRRGs increased in cluster A (**Figure 2B**; **Supplementary Figure S3B**). Furthermore, we observed notable variations in immune characteristics between patients in the two clusters. As depicted in **Figures 2F, G**, patients in cluster A displayed higher immune scores and immune cell infiltrations. For example, the infiltration levels of MDSCs, macrophages, monocytes, and Treg cells were elevated in cluster A, while only eosinophils exhibited reduced infiltration levels.

Distinct molecular biological functions were observed across the PCD clusters. GSVA (**Supplementary Figures S4A, B**) and GSEA results (**Supplementary Figures S4C, D**) revealed the activation of numerous tumor-associated biological processes and signaling pathways in cluster A. These processes encompassed epithelial-mesenchymal transition (EMT), cell proliferation (MYC targets, G2M checkpoints, E2F targets, cell cycle), and signaling pathways like WNT/β-Catenin, TGF-β, and PI3K/AKT. In contrast, cluster B exhibited the activation of several metabolism-associated biological processes, such as fatty acid metabolism and bile acid metabolism. These findings were corroborated by the results of the GO/KEGG analysis (**Supplementary Figures S5A–D**). In addition to disparities in tumor biological attributes, significant differences in various biological functions associated with PCD, such as apoptosis, necroptosis, and autophagy, were observed between the two clusters.

### Construction and evaluation of the prognostic predictive value of PCDI

**Figure 3:**
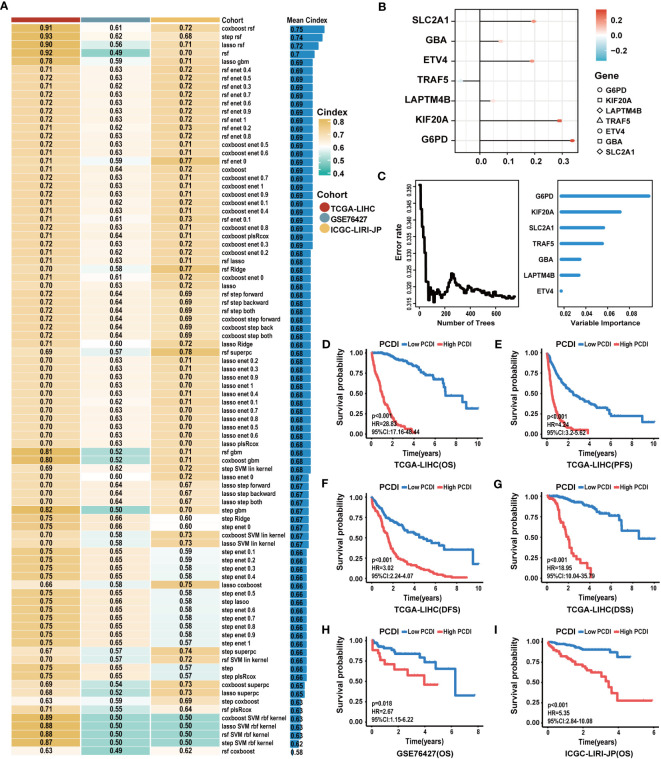
Construction of the PCDI based on an integrated framework for machine learning. **(A)** Combining 88 machine learning algorithms for prognostic models via 10-fold cross-validation and identifying the best one by C-index as the PCDI. **(B)** Determination of seven PCDI genes via the CoxBoost algorithm. **(C)** Determination of PCDI with minimal error and the importance of seven PCDI genes via the RSF algorithm. **(D–G)** Differences between patients in the high and low PCDI score groups for the OS, PFS, DFS, and DSS in TCGA-LIHC dataset. **(H, I)** Differences between patients in the high and low PCDI score groups for the OS in the GSE76427 and ICGC-LIRI-JP datasets.

Based on 87 prognostic PCD genes, we employed a machine learning algorithms integrated framework that combined 10 different machine learning algorithms through 10-fold cross-validation. The PCDI was constructed by integrating CoxBoost and RSF algorithms, which demonstrated the highest average C-index across three datasets among 88 algorithm combinations (**Figure 3A**). With the CoxBoost algorithm, we identified *GBA*, *G6PD*, *ETV4*, *KIF20A*, *LAPTM4B*, *TRAF5*, and *SLC2A1* as the seven most valuable PCD-related genes (**Figure 3B**; **Supplementary Table S8**). Furthermore, the RSF algorithm enhanced the reliability of this model (**Figure 3C**). We observed elevated expression levels of seven PCDI genes in HCC tissues (**Supplementary Table S6**), all of which were associated with an unfavorable prognosis (**Supplementary Table S7**; **Supplementary Figures S16A–G**). Concurrently, through GSEA, we detected that PCDI genes could trigger the activation of crucial tumor-associated biological processes, such as proliferation, invasion, and metastasis. Moreover, *TRAF5* and *SLC2A1* may be linked to immunological regulation, such as inflammation responses, and chemokine and T-cell receptor signaling pathways (**Supplementary Figures S6A–G**).

**Figure 4:**
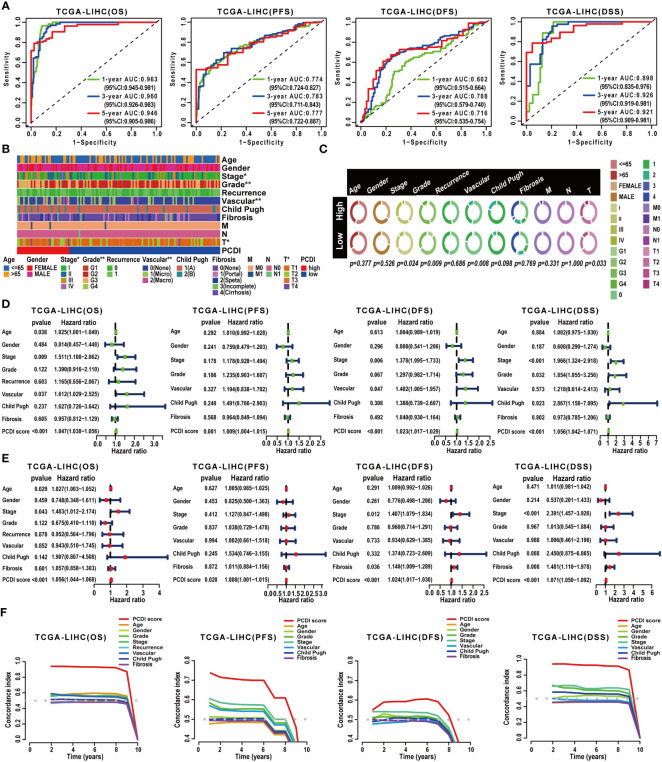
Validation of the prognostic predictive value of PCDI. **(A)** Evaluating the predictive accuracy of the PCDI for the OS, PFS, DFS, and DSS in TCGA-LIHC dataset with ROC curves. **(B, C)** Correlation analysis between clinicopathological characteristics and PCDI in TCGA-LIHC dataset. **(D)** Univariate Cox analysis revealing the impacts of PCDI and clinicopathological characteristics on OS, PFS, DFS, and DSS in TCGA-LIHC dataset. **(E)** Multivariate Cox analysis revealing the impacts of PCDI and clinicopathological characteristics on OS, PFS, DFS, and DSS in TCGA-LIHC dataset. **(F)** Comparing the prognostic predictive efficacy of PCDI and clinicopathological characteristics for the OS, PFS, DFS, and DSS in TCGA-LIHC dataset with C-index curves.

Subsequently, a comprehensive evaluation was performed for the prognostic predictive value of PCDI. In TCGA-LIHC dataset, survival curves demonstrated the PCDI could effectively predict the clinical outcomes of HCC patients, as indicated by survival metrics. Patients in the high PCDI score group suffered poor OS, PFS, DFS, and DSS compared to others (**Figures 3D–G**). ROC curves illustrated the accuracy of PCDI in prognostic prediction (**Figure 4A**). Notably, the highest accuracy was observed when utilizing the PCDI to predict OS, with the AUC values of 0.963 (95% CI: 0.945–0.981), 0.960 (95% CI: 0.926–0.983), and 0.946 (95% CI: 0.905–0.986) at 1 year, 3 years, and 5 years. Particularly, we found that PCDI scores for HCC patients in cluster A were significantly higher than those in cluster B, indicating congruence in terms of sample distribution (**Figures 2D, E**).

**Figure 5:**
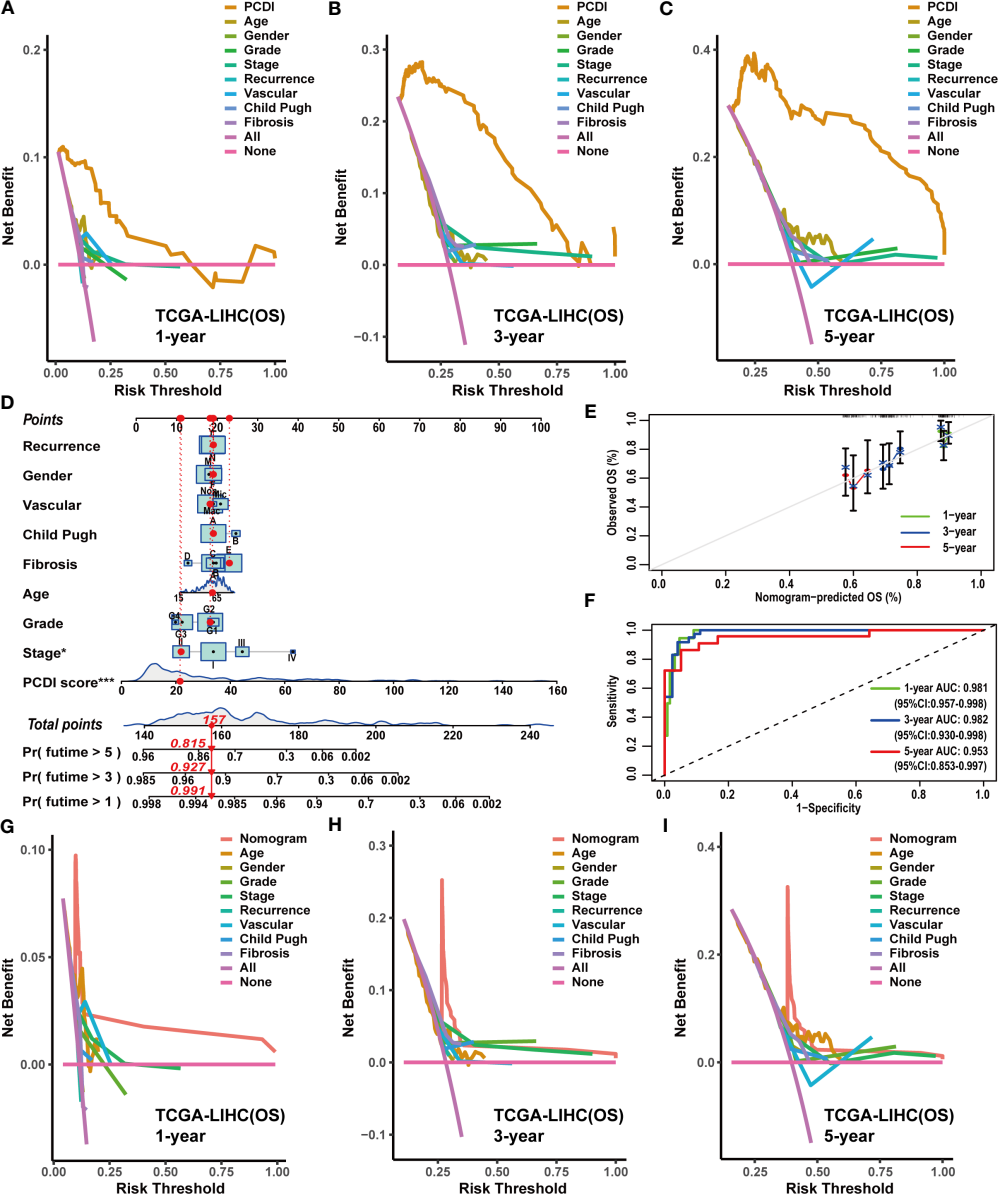
Construction and evaluation of the nomogram based on PCDI and clinicopathological characteristics. **(A–C)** Comparing the prognostic predictive efficacy of PCDI and clinicopathological characteristics for OS with DCA curves in TCGA-LIHC dataset. **(D)** Construction of a nomogram with PCDI and clinicopathological characteristics for predicting OS in TCGA-LIHC dataset. **(E)** Evaluating the predictive accuracy of a nomogram for the OS with calibration curves in TCGA-LIHC dataset. **(F)** Evaluating the predictive accuracy of a nomogram for the OS with ROC curves in TCGA-LIHC dataset. **(G–I)** Comparing the predictive efficacy of nomogram and clinicopathological characteristics for the OS in TCGA-LIHC dataset with DCA curves.

**Figure 6:**
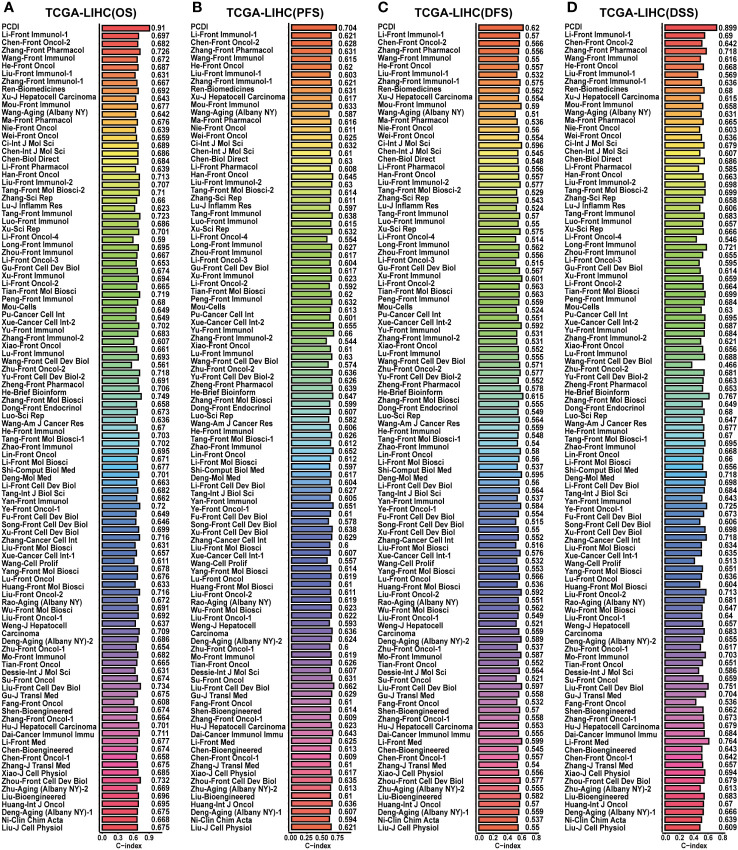
Comparison of the predictive value between PCDI and other models in TCGA-LIHC dataset. **(A)** Comparing the prognostic predictive efficacy of PCDI and other published models for OS by C-index analysis. **(B)** Comparing the prognostic predictive efficacy of PCDI and other published models for PFS by C-index analysis. **(C)** Comparing the prognostic predictive efficacy of PCDI and other published models for DFS by C-index analysis. **(D)** Comparing the prognostic predictive efficacy of PCDI and other published models for DSS by C-index analysis.

Afterward, we conducted a correlation analysis between PCDI and clinicopathological attributes. The PCDI exhibited a significant association with the advanced clinical stage, T stage, pathological grade, and vascular invasion status among HCC patients (**Figures 4B, C**). Independent prognostic analysis revealed PCDI as an independent risk factor for the OS, PFS, DFS, and DSS in HCC patients (**Figures 4D, E**). Through C-index and DCA curves, we observed that the PCDI exhibited superior predictive performance compared to other clinicopathological attributes in predicting OS, PFS, DFS, and DSS (**Figures 4F**, **5A–C**; **Supplementary Figure S7A–I**). Additionally, when compared with 102 published prognostic predictive models, a C-index analysis affirmed the superiority of PCDI (**Figures 6A–D**).

Finally, the prognostic predictive value of PCDI was validated in the GSE76427 and ICGC-LIRI-JP datasets. Survival curves substantiated the capacity of PCDI to effectively predict the clinical outcomes of HCC patients in both datasets, indicating a worse OS in patients with higher PCDI scores (**Figures 3H, I**). In the GSE76427 dataset, ROC curves presented the AUC values of PCDI as 0.629 (95% CI: 0.517–0.761), 0.631 (95% CI: 0.531–0.772), and 0.659 (95% CI: 0.556–0.790) at 1 year, 3 years, and 5 years in predicting OS (**Supplementary Figure S8A**), and in the ICGC-LIRI-JP dataset, the values were 0.757 (95% CI: 0.630–0.890), 0.726 (95% CI: 0.661–0.858), 0.692 (95% CI: 0.549–0.818) (**Supplementary Figure S9A**). In both datasets, Chi-square analysis revealed a significant correlation between PCDI and advanced clinical stages (**Supplementary Figures S8B, C**, **S9B, C**). Independent prognostic analysis demonstrated that the PCDI served as an independent risk factor for worse outcomes in HCC (**Supplementary Figures S8D, E**, **S9D, E**). C-index and DCA curves indicated excellent prognostic predictive performance of the PCDI in both datasets (**Supplementary Figures S8F–I**, **S9F–I**). When compared with published predictive models, the PCDI consistently demonstrated exemplary performance (**Supplementary Figures S10A, B**).

### Construction and evaluation of the predictive efficacy of clinical nomograms

Owing to the remarkable prognostic predictive value of PCDI, we developed a clinical nomogram to facilitate the utilization of PCDI. In TCGA-LIHC dataset, the PCDI was integrated with other clinicopathological factors to establish a clinical nomogram for predicting the OS of patients. As shown in **Figure 5D**, the PCDI score emerged as a significant variable in the clinical nomogram. Calibration and ROC curves indicated the exceptional predictive efficacy of this nomogram (**Figures 5E, F**). Furthermore, DCA curves validated the superior predictive efficacy of this clinical nomogram for OS compared to other clinicopathological factors (**Figures 5G–I**).

Subsequently, we applied a similar method to construct clinical nomograms in the GSE76427 and ICGC-LIRI-JP datasets. In the GSE76427 dataset, the PCDI score was the significant variable in the clinical nomogram (**Supplementary Figure S11A**), and a similar result was observed in the ICGC-LIRI-JP dataset (**Supplementary Figure S12A**). Calibration and ROC curves clearly demonstrated the favorable predictive performance of these clinical nomograms in predicting OS (**Supplementary Figures S11B, C**, **S12B, C**). Additionally, DCA curves affirmed the nice predictive performance of clinical nomograms for OS (**Supplementary Figures S11D–F**, **S12D–F**).

### Clarifying the characteristics of immunology and biological function based on the PCDI score in HCC

**Figure 7:**
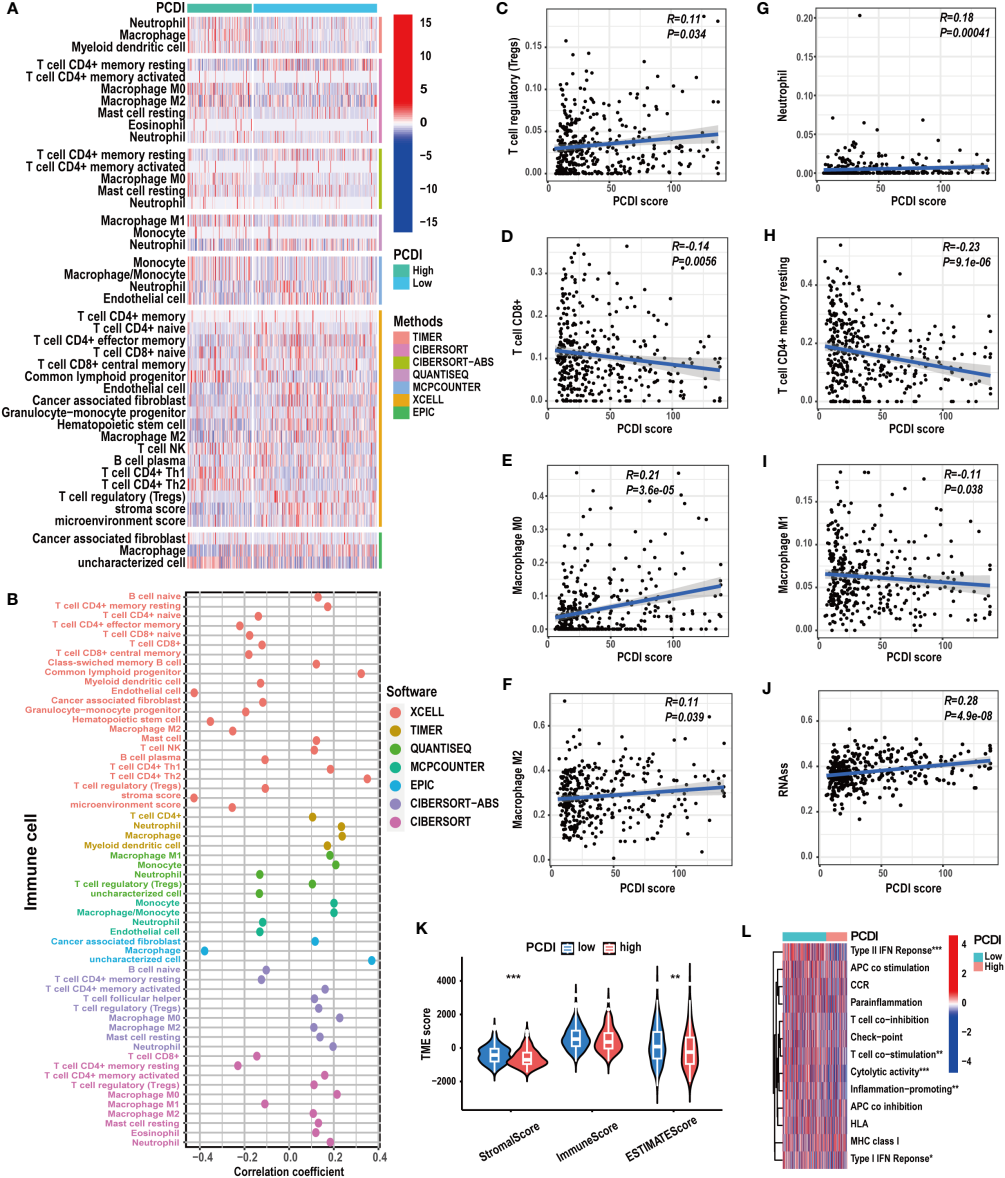
The correlation between immunological characteristics and PCDI. **(A, B)** Different immune cell infiltration patterns between the high and low PCDI score groups. **(C-I)** Correlation analysis of the immune cell infiltration levels and PCDI scores. **(J)** Correlation analysis of the stemness scores and PCDI scores. **(K)** Different TME scores between the high and low PCDI score groups. **(L)** Different immune function statuses between the high and low PCDI score groups. (*p < 0.05; **p < 0.01; ***p< 0.001).

To explore the correlation between PCDI and immunological features in HCC patients, we conducted a comprehensive investigation. Using multiple immunological algorithms such as TIMER, CIBERSORT, and XCELL, we observed notable differences in immune cell infiltration levels between the high and low PCDI score groups (**Figures 7A, B**). Furthermore, we found that the infiltration levels of Treg cells, neutrophils, and M0 and M2 macrophages exhibited a significant positive correlation with PCDI scores, whereas CD8^+^ T cells, CD4^+^ T cells, and M1 macrophages displayed a significant negative correlation (**Figures 7C–I**). Employing the ESTIMATE algorithm, we found that the stromal and estimate scores of patients with higher PCDI scores were significantly decreased compared to those with lower PCDI scores. However, no statistically significant differences were observed in the immune scores between these two groups (**Figure 7K**). Additionally, we noticed that, compared to patients with lower PCDI scores, patients with higher PCDI scores exhibited significant suppression of type I/II IFN responses, T-cell co-stimulation, cytotoxic responses, and proinflammatory processes (**Figure 7L**). These findings indicated a potential suppressive immune microenvironment in the high PCDI score group and enhanced stromal cell infiltration in the TME of the low PCDI score group. Furthermore, a significant positive correlation was detected between stemness score and PCDI score, suggesting the potential presence of active cancer stem cells in the TME of patients with higher PCDI scores (**Figure 7J**).

Subsequently, we compared the biological functional attributes between HCC patients in the high and low PCDI score groups. Through GSVA (**Supplementary Figures S13A, B**) and GSEA (**Supplementary Figures S13C, D**), we observed a significant activation of tumor-associated biological processes such as EMT, cell proliferation (MYC targets, E2F targets, G2M checkpoints, and cell cycle), and signaling pathways like WNT/β-catenin and PI3K/AKT/MTOR pathways in the high PCDI score group. Conversely, metabolic-associated processes, such as fatty acid metabolism and bile acid metabolism, were notably activated in the low PCDI score group. Furthermore, between these two groups, GO/KEGG analysis (**Supplementary Figures S14A–D**) revealed notable differences in various oncological biological functions and numerous cellular processes associated with cell replication, such as nuclear division and chromosomal disjunction regulation. Additionally, various metabolic-related processes exhibited distinct patterns.

### Clarifying ICG expression patterns and gene mutation statuses based on the PCDI score in HCC

**Figure 8:**
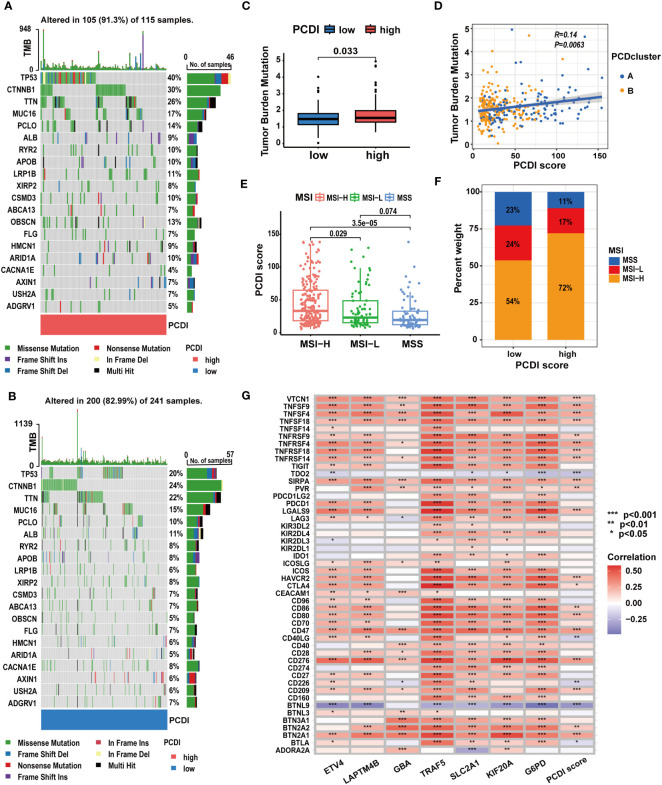
The correlation between mutation characteristics, ICG expression patterns, and PCDI. **(A, B)** Different genetic mutation characteristics between the high and low PCDI score groups. **(C)** Different TMB levels between the high and low PCDI score groups. **(D)** Correlation analysis of TMB, PCD clusters, and PCDI scores. **(E)** Correlation analysis of MSI statuses and PCDI scores. **(F)** Distribution of patients with different MSI statuses across PCDI score groups. **(G)** Correlation analysis of ICG expression levels and PCDI. (*p < 0.05; **p < 0.01; ***p< 0.001).

We explored the correlation between PCDI and gene mutation statuses along with ICG expression patterns. We found a higher frequency of gene mutations in the high PCDI score group. Missense mutations were the predominant mutation type observed. *TP53* emerged as the most frequently mutated gene in the high PCDI score group, displaying the greatest disparity in mutation frequency between the two groups. Moreover, *CTNNB1* mutations were most prevalent in the low PCDI score group (**Figures 8A, B**). Further analysis indicated an obvious increase in TMB levels among patients in the high PCDI score group. Concurrently, patients with higher TMB levels were predominantly classified as cluster A (**Figures 8C, D**). Similarly, we observed a positive correlation between MSI levels and PCDI scores. Patients with high MSI levels were primarily clustered in the high PCDI score group (**Figures 8E, F**). Moreover, correlation analysis of PCDI and ICG expression patterns revealed that the expression of the majority of ICGs exhibited a significant positive correlation with PCDI scores (**Figure 8G**; **Supplementary Table S9**).

### Evaluation and valuation of the predictive value of PCDI in immunotherapy responses

Considering the correlation between PCDI and TMB, MSI, and ICG expression patterns, we examined the predictive value of PCDI in patients’ responses to immunotherapy.

**Figure 9:**
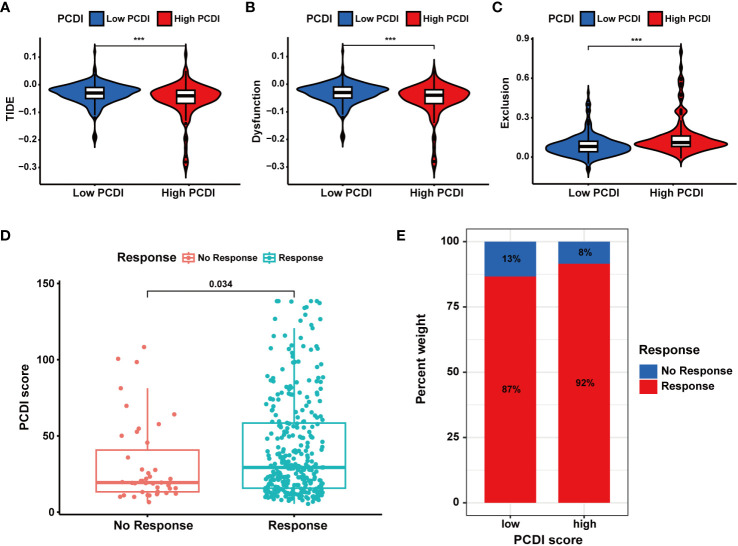
Evaluation of the predictive value of PCDI in immunotherapy responses based on TIDE algorithms. **(A)** Different TIDE scores between the high and low PCDI score groups. **(B)** Different dysfunction scores between the high and low PCDI score groups. **(C)** Different exclusion scores between the high and low PCDI score groups. **(D)** Correlation analysis of immunotherapy response statuses and PCDI scores. **(E)** The distribution of patients with different immunotherapy response statuses across the PCDI score groups.

In TCGA-LIHC dataset, we calculated the TIDE, dysfunction, and exclusion scores for HCC patients through the TIDE algorithm. We found the TIDE and dysfunction scores exhibited a notable reduction in the high PCDI score group, while the exclusion score demonstrated an increase (**Figures 9A–C**). These results suggested patients with higher PCDI scores could respond to immunotherapy easily. Further analysis indicated a significant relationship between higher PCDI scores and an increased response rate to immunotherapy. More patients responding to immunotherapy were found in the high PCDI score group (**Figures 9D, E**). These results reaffirmed our earlier findings, indicating that patients with higher PCDI scores were more responsive to immunotherapy. PCDI could be employed for immunotherapy response prediction in HCC patients.

**Figure 10:**
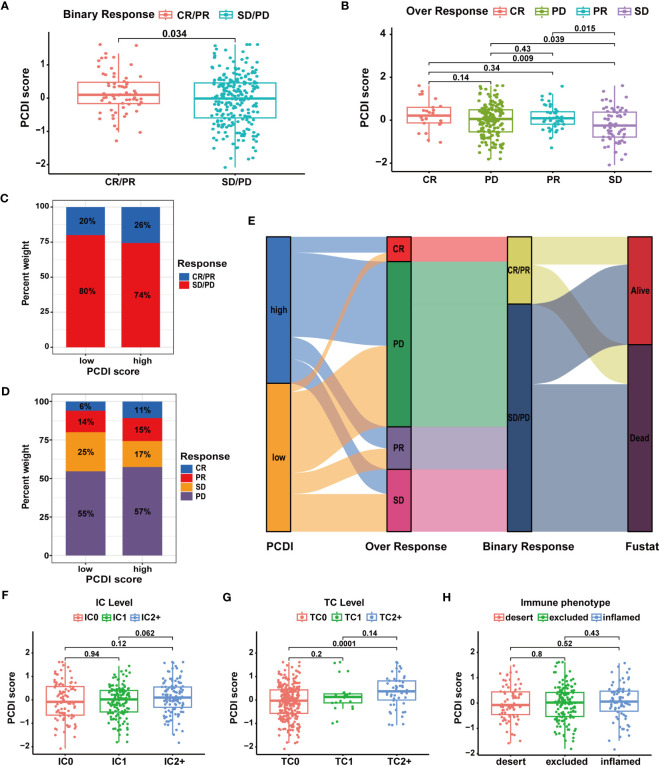
Validation of the predictive value of PCDI for immunotherapy response in the IMvigor210 cohort. **(A, B)** Correlation analysis of immunotherapy response statuses and PCDI scores. **(C, D)** The distribution of patients with different immunotherapy response statuses across the PCDI score groups. **(E)** The distribution of patients with different immunotherapy responses and OS statuses across the PCDI score groups. **(F)** Correlation between the levels of immune cells and PCDI scores. **(G)** Correlation between the levels of tumor cells and PCDI scores. **(H)** Correlation between TME characteristics and PCDI scores.

Subsequently, we analyzed multiple immunotherapy cohorts to further validate the predictive efficacy of PCDI for immunotherapy responses. In the IMvigor210 cohort, we found a higher PCDI score was significantly associated with a better response rate to immunotherapy, and more patients responding to immunotherapy were in the high PCDI score group (**Figures 10A–E**). Furthermore, we observed that the median levels of immune cell infiltration were elevated in patients with higher PCDI scores, but there was no significant difference. Additionally, there was no significant correlation between immune microenvironment statuses and PCDI scores, while tumor cell infiltration levels were positively associated with PCDI scores (**Figures 10F–H**). Moreover, in the GSE176307, Checkmate, GSE179351, GSE103668, and GSE78220 cohorts, patients in the higher PCDI score group demonstrated a greater likelihood of responding to immunotherapy (**Supplementary Figures S15A–G**); and in the GSE35640 and GSE120644 cohorts, patients in the low PCDI score group were more responsive to immunotherapy (**Supplementary Figures S15I, J**). In the GSE91061 cohort, PCDI appeared to have no association with the immunotherapy responses (**Supplementary Figure S15H**). Overall, the PCDI can effectively predict patients’ responses to immunotherapy, and it can guide immunotherapy for patients based on PCDI scores. Particularly for HCC patients, those with higher PCDI scores could be better candidates for immunotherapy.

Additionally, as shown in **Supplementary Figures S15K, L**, PCDI can also predict the responses of HCC patients to sorafenib and TACE treatments. Patients in the low PCDI score group were more likely to respond to sorafenib and TACE therapies, suggesting that patients with lower PCDI scores could be better candidates for these treatments.

### Evaluation of the predictive value of PCDI in chemotherapy sensitivity for HCC

**Figure 11:**
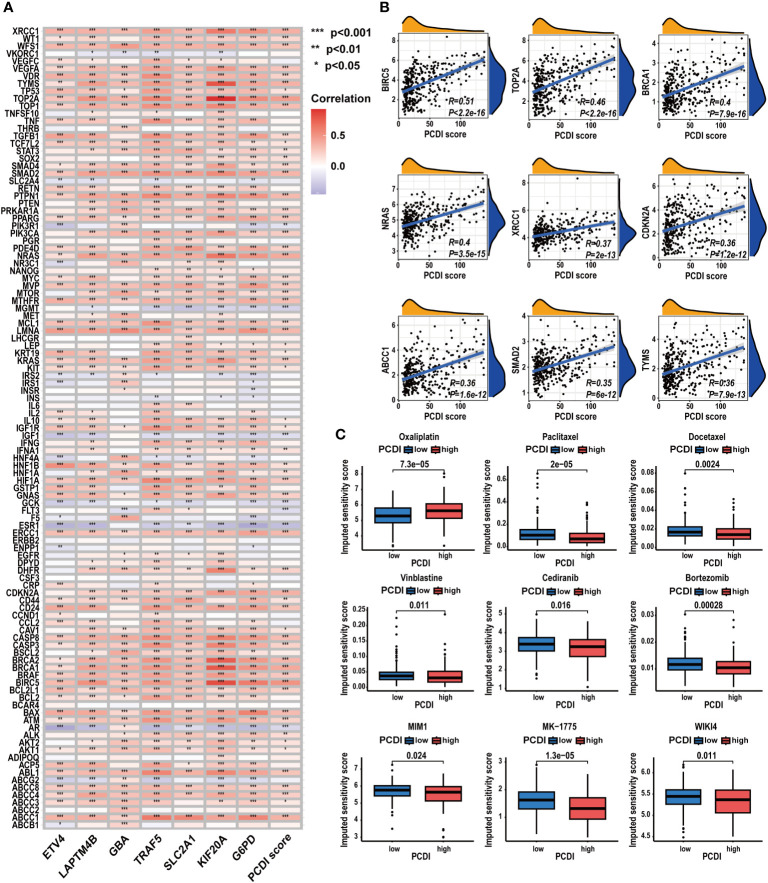
Correlation between CRRG expression patterns, chemotherapeutic drug sensitivities, and PCDI. **(A)** Correlation analysis of CRRG expression levels and PCDI. **(B)** Correlation analysis of multiple CRRG expression levels and PCDI scores. **(C)** Different drug sensitivities between the high and low PCDI score groups. (*p < 0.05; **p < 0.01; ***p< 0.001).

We further investigated the predictive value of the PCDI in chemotherapy. As depicted in **Figure 11A**, we found a significant positive correlation between the PCDI scores and the expression levels of most CRRGs. The results suggested the PCDI could be used for assessing the drug resistance of HCC patients, and PCDI genes may represent promising targets for overcoming chemotherapeutic resistance in HCC (**Supplementary Table S10**). **Figure 11B** visualizes the first nine CRRGs exhibited a positive correlation with the PCDI score. We employed the “OncoPredict” package to further validate the capability of PCDI in drug sensitivity prediction. As illustrated in **Figure 11C**, in the low PCDI score group, the imputed sensitivity score of oxaliplatin was significantly reduced, indicating a heightened sensitivity in patients with lower PCDI scores. Conversely, in the high PCDI score group, several drugs such as paclitaxel, docetaxel, vinblastine, cediranib, and bortezomib displayed lower imputed sensitive scores, implying a potential increase in sensitivity to these drugs in these patients.

### Comprehensive analysis of the PCDI genes

To acquire a deeper understanding of the PCDI, we performed a comprehensive analysis of the PCDI genes in HCC.

**Figure 12:**
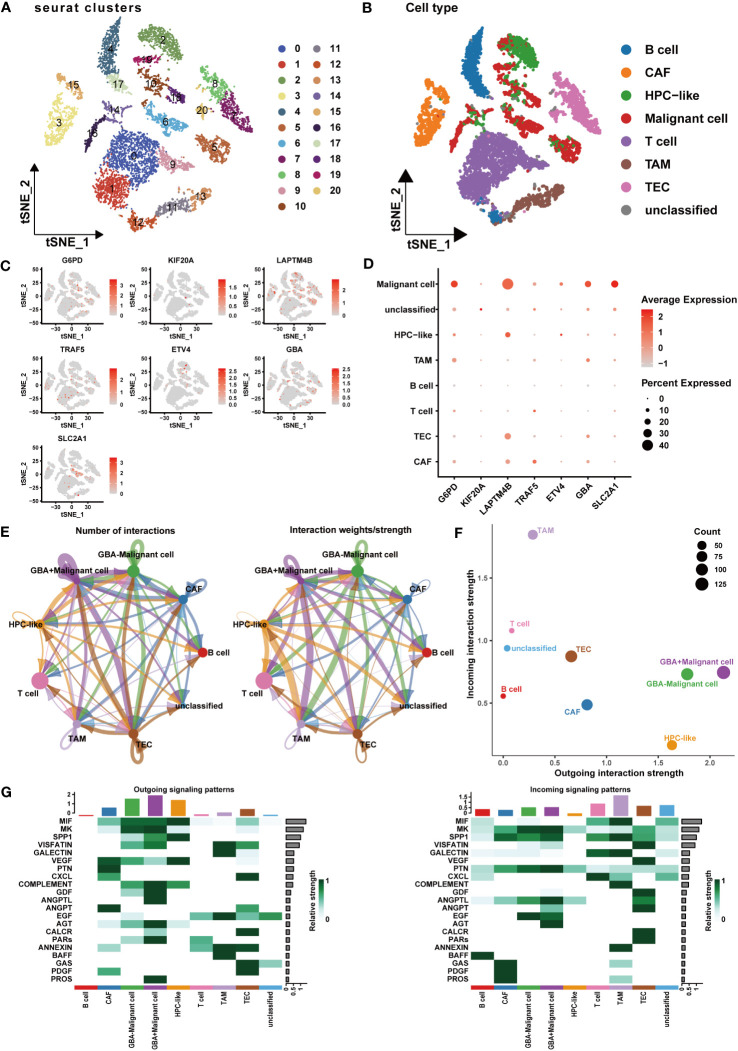
Expression patterns and cellular communication characteristics of the PCDI genes at single-cell level. **(A, B)** The results of cell clustering and annotation for the GSE125449 dataset. **(C, D)** The expression patterns of seven PCDI genes in different cell populations. **(E, F)** The cellular communication network among different cell populations. **(G)** The activated state of specific pathways in different cell populations.

At the single-cell level, we investigated the expression patterns and cellular communication characteristics of PCDI genes. Employing the t-SNE method for cluster analysis, we identified 21 cell clusters, which were annotated as eight primary cell populations (**Figures 12A, B**). Subsequently, we explored the expression patterns of seven PCDI genes across different cell populations (**Figures 12C, D**). We observed stable expression of PCD genes in malignant cells, with *LAPTM4B*, *G6PD*, *SLC2A1*, and *GBA* exhibiting the highest expression levels. Notably, besides malignant cells, PCDI genes are also expressed in immune and stromal cells. *G6PD* is mainly expressed in TAMs, *LAPTM4B* is predominantly expressed in tumor endothelial cells (TECs), *GBA* is expressed in both cell populations, and *TRAF5* is primarily expressed in cancer-associated fibroblasts (CAFs). Moreover, we conducted a cellular communication analysis. Given the limited research on *GBA* in HCC and its high expression level in malignant cells and suppressive immune cells, we selected it as the focal point of this analysis. We divided malignant cells into two groups: *GBA+* and *GBA−*, based on their *GBA* expression levels (*GBA+* indicating high expression, *GBA−* indicating low expression). We then compared the cellular communication characteristics between the two groups. The communication network among all cell populations is displayed in **Figures 12E, F**. Among all cell populations, malignant cells exhibited the most extensive cell communication and showed the highest signal output intensity. TAMs, TECs, CAFs, and HPCs exhibited similar numbers of cellular interactions, with TAMs demonstrating the highest signal input strength. Notably, *GBA+* malignant cells exhibited more extensive cell communication in terms of both quantity and strength. For specific cellular communication pathways, *GBA*+ malignant cells exhibited higher activation levels in pathways such as SPP1, GDF, ANGPTL, PARs, and PROS (**Figure 12G**). This suggested more active biological processes in *GBA+* malignant cells, including cell proliferation, invasion, metastasis, angiogenesis, and inflammatory responses.

At the protein level, we explored the expression patterns and prognostic correlations of the PCDI genes. Utilizing the proteome dataset PDC-000198, we observed a significant upregulation in the expression of *GBA*, *G6PD*, and *KIF20A* in HCC tissue, which was associated with unfavorable clinical outcomes. Although *SLC2A1* and *TRAF5* exhibited no significant expression difference between HCC and adjacent tissues, they still displayed an association with a poor prognosis. Unfortunately, data for *LAPTM4B* and *ETV4* were not available in this dataset (**Supplementary Table S11**; **Supplementary Figures S16H–L**).

**Figure 13:**
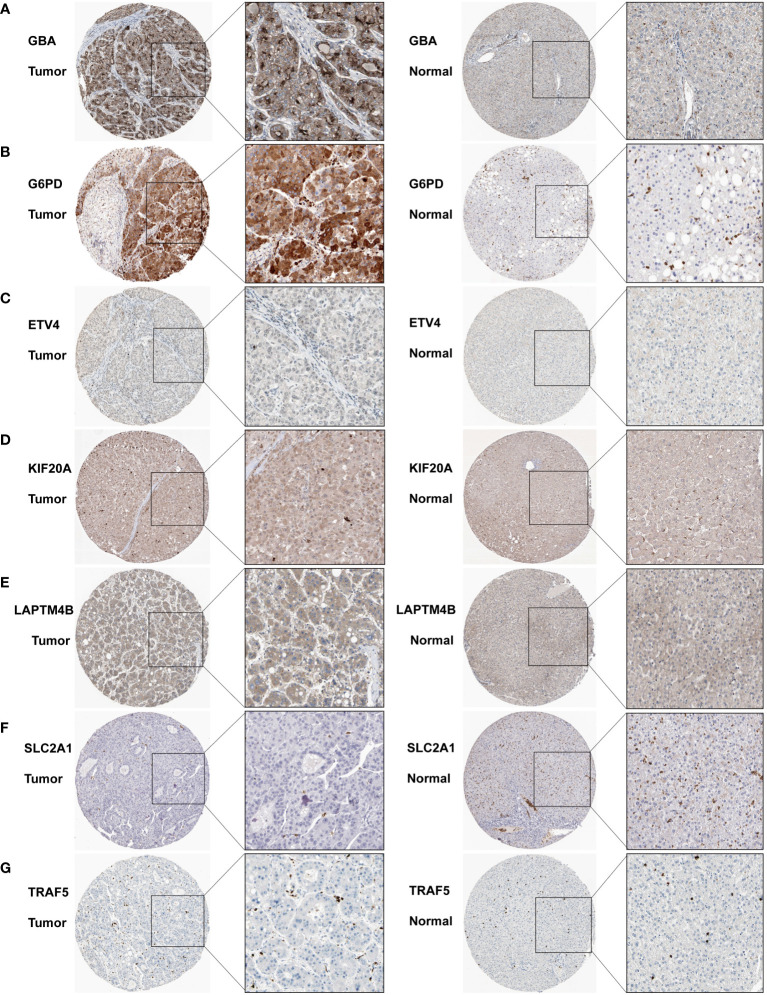
Immunohistochemistry results for the PCDI genes. **(A–G)** Different protein expression levels of PCDI genes between tumor and normal tissues in the HPA database.

We subsequently acquired IHC data for PCDI genes from the HPA database. Among them, *GBA*, *G6PD*, and *KIF20A* demonstrated remarkably elevated expression levels in HCC tissue. Similarly, *SLC2A1* and *TRAF5* also displayed a modest difference between HCC tissue and normal tissue. Additionally, *LAPTM4B* exhibited notably heightened expression in HCC tissue, while *ETV4* exhibited slightly higher expression in HCC tissue (**Figures 13A–G**).

### Functional evaluation of the PCDI genes

We then aimed to provide experimental evidence elucidating the involvement of PCDI genes in HCC. Building upon prior findings, a sequence of functional investigations focused on GBA was undertaken.

**Figure 14:**
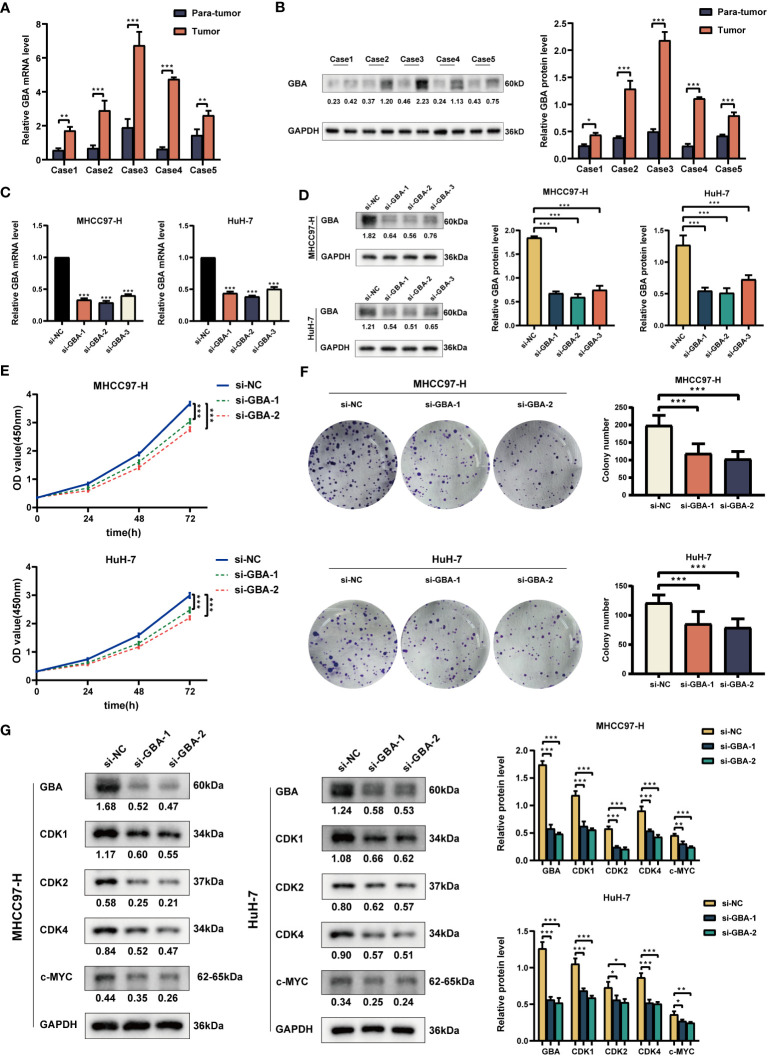
Experimental validation of GBA on proliferation. **(A)** Relative expression of GBA in HCC tumor tissues and para-tumor tissues at the mRNA level. **(B)** Expression of GBA in HCC tumor tissues and para-tumor tissues at the protein level. **(C, D)** Verification of GBA knockdown efficiency with siRNA at the mRNA and protein levels in MHCC97H and HuH-7 cells. **(E, F)** Effects of GBA knockdown on the proliferation capability of both cell lines detected with CCK-8 and colony formation assays. **(G)** Effects of GBA knockdown on cell cycle-associated markers in both cell lines detected by WB. (^*^ *p* < 0.05; ^**^ *p* < 0.01; ^***^ *p* < 0.001.).

**Figure 15:**
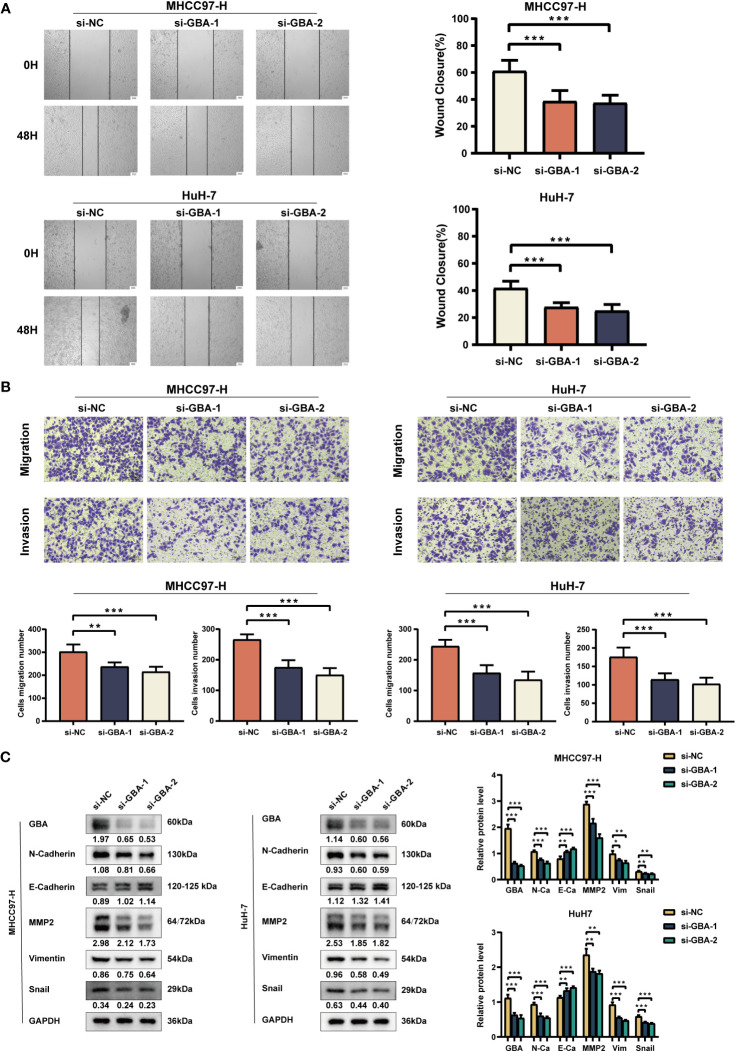
Experimental validation of GBA on invasion and migration. **(A)** Effects of GBA knockdown on the migration capability of both cell lines detected with a wound healing test. Scale bar: 100 µm (×40). **(B)** Effects of GBA knockdown on the migration and invasion capability of both cell lines detected with a Transwell assay. Scale bar: 100 µm (×200). **(C)** Effects of GBA knockdown on EMT-associated markers in both cell lines detected by WB. (^*^ *p* < 0.05; ^**^ *p* < 0.01; ^***^ *p* < 0.001.).

As shown in **Figures 14A, B**, both qRT-PCR and WB analyses consistently revealed a significant upregulation of GBA in tumor tissues. Afterward, we downregulated *GBA* expression levels in MHCC97H and HuH-7 through transfection of siRNAs. *GBA* knockdown was validated at both the mRNA and protein levels, and three distinct siRNAs, si-NC (control), si-*GBA*-1, and si-*GBA*-2, were selected for subsequent experiments (**Figures 14C, D**). The results of the CCK-8 assay and colony formation test revealed *GBA* knockdown significantly suppressed the proliferative capacity of MHCC97H and HuH-7 cells (**Figures 14E, F**). Simultaneously, WB analysis demonstrated *GBA* knockdown substantially reduced the expression levels of *CDK1*, *CDK2*, *CDK4*, and *c-MYC* in both two HCC cell lines (**Figure 14G**). These results indicated the integral role of *GBA* in the regulation of the cell cycle and tumor proliferation. Furthermore, our results suggest that *GBA* may also be involved in the invasive processes of the tumor. The wound-healing test revealed that the downregulation of *GBA* significantly attenuated the scratch closure rates of MHCC97H and HuH-7 cells (**Figure 15A**), suggesting a reduced migratory capacity of HCC cells. The Transwell assay further validated that *GBA* knockdown resulted in a diminished migratory and invasive capacity of HCC cells (**Figure 15B**). WB analysis illustrated that in both MHCC97H and HuH-7 cell lines, *GBA* knockdown notably decreased the expression levels of *N-cadherin*, *Vimentin*, *Snail*, and *MMP2*. Conversely, the expression levels of *E-cadherin* increased with the downregulation of *GBA* (**Figure 15C**). These findings suggested *GBA* was involved in the EMT in HCC cells, thereby enhancing their invasive and metastatic potential.

## Discussion

Despite notable advancements in HCC therapies, the clinical prognosis for patients remains unsatisfactory. Surgical interventions, such as resection and transplantation, are the optimal therapeutic strategies for early-stage HCC patients. However, over half of them experience a relapse within 5 years following a hepatectomy. While the recurrence rate is lower for liver transplant recipients, the widespread adoption of liver transplantation is constrained by the limited availability of donors (1, 39). For the majority of patients diagnosed with advanced-stage HCC, systemic treatments are the primary therapeutic approach. The application of TKIs and ICIs represents a significant transformation in the current systemic treatment for HCC. However, these treatments have only resulted in modest improvements in survival time, ranging from 1.2 to 5.8 months, which falls short of expectations (40–45). This may be attributed to the limited success of personalized treatment owing to tumor heterogeneity. Although clinical staging systems provide a foundation for HCC management, they are incapable of assessing the molecular biological characteristics of patients. This limitation is a significant hindrance to the implementation of personalized treatments for HCC. Thus, there is an imperative requirement to identify potent biomarkers as complementary tools to existing staging systems for guiding therapeutic decisions, which could elevate the level of personalized treatment and enhance the clinical management of HCC, thus improving the prognosis.

Distinct from accidental cell death, PCD is a complex process characterized by intricate regulation and diverse operational patterns. Accumulated evidence has implicated various cell death modes as pivotal hallmarks of tumorigenesis, potentially serving as a theoretical foundation for innovative anticancer strategies (8). In this study, we presented a comprehensive examination of the correlation between 14 distinct PCD modes and the clinical characteristics along with the biological patterns of HCC for the first time. Initially, we investigated the expression patterns of PCD-related genes in HCC. We identified 756 differentially expressed PCD-related genes, with 721 of them exhibiting increased expression in tumor tissues. Among these genes, we further identified 87 prognostic PCD genes, with 85 of them associated with an unfavorable prognosis. These findings indicated a potential role for PCD-related genes in HCC. Among the 87 prognostic-related PCD genes, CNVs were frequently observed, and approximately one-third of patients experienced mutations of these genes. Indeed, there is some evidence suggesting that mutations in specific genes could participate in PCD regulation and influenced tumorigenesis. For example, mutations in the *TP53* gene could disrupt various PCD pathways, playing a crucial role in HCC progression (46).

Subsequently, we identified two PCD clusters in HCC patients. These two PCD clusters exhibited notable differences in sample distribution, clinical attributes, and biological features. HCC patients in cluster A displayed more severe clinical manifestations, such as advanced clinical stage, pathological grade, and poor prognosis. In addition to higher expression levels of PCD-related genes, HCC patients in cluster A also demonstrated elevated expression of ICGs and CRRGs compared to those in cluster B. Drug resistance mechanisms in HCC have been categorized into seven types, encompassing drug uptake and export, drug metabolism, alterations in drug targets, DNA repair, disruption in apoptosis/survival signals, adaptation to the TME, and phenotypic transition. These mechanisms can elucidate the roles of most CRRGs in regulating drug sensitivity in HCC (34). Similarly, ICGs could be categorized into three types: tumor cell dominant, immune cell dominant, and balanced type. HCC patients with higher expression of ICGs exhibited a favorable prognosis and were more likely to benefit from immunotherapy (33). These findings indicated a potential association between treatment responses for HCC patients and the expression patterns of ICGs and CRRGs, which could cause variable treatment responses. Despite patients in cluster A exhibiting higher immune scores and enhanced immune cell infiltration, the presence of cells including MDSCs, macrophages, monocytes, and Treg cells suggested the existence of an immunosuppressive microenvironment (47). In addition, we observed a significant activation of numerous tumor-associated biological functions and pathways in cluster A, such as EMT, cell proliferation (MYC targets, G2M checkpoints, E2F targets, and cell cycle), WNT/β-catenin, TGF-β, and PI3K/AKT signaling pathways. The characteristics of these HCC patients, including an unfavorable prognosis, increased proliferation, heightened invasiveness, and pathway activation, align with the proliferative subtype in the classical classification of HCC. Additionally, the activation of the WNT/β-catenin pathway has been defined as a hallmark of the nonproliferative subtype of HCC, which correlates with enhanced immune infiltration (48, 49). Notably, these features were also observed in HCC patients in cluster A. Our findings substantiate the close association between PCD clusters and the clinical and biological characteristics of HCC patients. We posited that focusing on PCD could offer a novel perspective for comprehending the pathogenesis, evolution, and treatment of HCC.

Afterward, we constructed the well-performing PCDI model, which could serve as a tool for prognostic prediction and therapeutic guidance in HCC. Indeed, with the advancements in gene sequencing and bioinformatics techniques, there has been an exponential increase in genomic and molecular data from both tissues and single cells. This abundance of data has led to the identification of numerous gene signatures (referred to as prognostic models) similar to the PCDI model. These gene signatures could be used to assess patients at a molecular level and group them based on shared phenotypes, such as clinical and molecular biological characteristics and responses to specific treatments. Thus, these signatures can assist clinicians in patient risk stratification and screening potential beneficiaries of certain treatments. For example, He et al. reported a coagulation pathway subtype in HCC with distinct immunological and prognostic features. They further developed a coagulation-related gene risk score to predict patient prognosis and treatment responses (50). Zeng et al. developed a hypoxia-driven gene signature for predicting and improving outcomes for HCC patients (51). Liu et al. established a prognostic model for HCC with cuproptosis-related genes and the RSF algorithm, which was used for patient risk stratification and treatment beneficiary selection (52). In diseases with complex etiologies and heterogeneity, such as HCC, these gene signatures, which were composed of multiple genes, demonstrated greater reliability compared to biomarkers such as *AFP*, *PD-L1*, and TMB (53). However, most gene signatures were constructed using a single algorithm, typically a regression algorithm (e.g., LASSO regression) or a machine learning algorithm (e.g., the RSF algorithm). This often resulted in decreased stability and generalizability, manifesting as a significant decrease in accuracy when tested on validation or external datasets. Furthermore, these studies frequently lack lateral comparisons among prognostic models, hindering the further validation of their predictive efficacy. These limitations may compromise the ability of most gene signatures to accurately predict and guide personalized treatment for HCC patients. In this study, we employed a novel framework that integrated 10 machine learning algorithms and generated 88 prognostic models via algorithmic combinations. The model composed of a CoxBoost and RSF algorithmic combination was identified as the best one, referred to as the PCDI. Compared to singular algorithms, the integration and combination of multiple algorithms could effectively reduce the dimensionality of variables, optimize stability and generalizability, and thereby enhance the performance of prognostic models. Furthermore, through extensive lateral comparisons, the superior performance of the PCDI model has been further substantiated. This also highlighted the potential utility of integrating and combining multiple algorithms in developing high-performance gene signatures.

In this study, the PCDI was comprised of seven genes: *GBA*, *G6PD*, *ETV4*, *KIF20A*, *LATPM4B*, *TRAF5*, and *SLC2A1*. *G6PD*, an essential rate-limiting enzyme of the pentose phosphate pathway, exhibits notable upregulation in HCC patients. *G6PD* was reported as a promoter in tumor growth, invasion, and metastasis, correlating with a poor prognosis. Concurrently, *G6PD* suppresses ferroptosis by downregulating *POR* expression. Targeting *G6PD* could potentially inhibit the progression of HCC (54, 55). *ETV4* expression was upregulated in HCC tissues, involved in the modulation of numerous oncogenes, proteins, and signaling pathways, thereby contributing to HCC progression (56). Increased *KIF20A* expression has been observed in mouse HCC models and could promote tumor proliferation. Knockdown of *KIF20A* in human HCC cell lines could also suppress cell growth and enhance their sensitivities to sorafenib and cisplatin (57, 58). *LATPM4B*, which was overexpressed in HCC, induced malignant behaviors, including proliferation, migration-invasion, and stem cell phenotypes (59, 60). *TRAF5* enhanced the ability of HCC in proliferation and invasion-metastasis. Reduction of *TRAF5* could induce necroptosis, thereby impeding HCC progression (61, 62). *SLC2A1* expression was upregulated in numerous solid tumors, including HCC. *SLC2A1* could promote HCC progression, and suppressing *SLC2A1* could induce immunogenic cell death in HCC (63, 64). Currently, few studies have addressed the role of *GBA* in HCC. One study suggested that *GBA* may be implicated in the antineoplastic activity of artemisinin against HCC (65). These results illuminated the complex involvement of the PCDI genes in HCC. In our study, we also performed a multilevel investigation of PCDI genes based on single-cell transcriptomic data, transcriptomic data, proteomic data, and IHC data. At the single-cell level, we provided a possible explanation for the correlation between PCDI and biological characteristics in patients with HCC. PCDI genes are primarily expressed in malignant cells and are also observed in certain immune and stromal cells such as TAMs and CAFs. These findings suggested the presence of massive active tumor cells and a suppressive immune microenvironment in the tumor tissues of patients with high PCDI scores. This was consistent with the TME characteristics of patients in the high PCDI score group. At the mRNA and protein levels, our findings further validated the oncogenic potential of PCDI genes. Furthermore, we investigated the role of *GBA* in HCC. Single-cell analysis revealed that *GBA* is predominantly expressed in malignant cells, TAMs, and TECs. Concurrently, *GBA* promoted the formation of cellular communication between malignant cells and other cells, particularly between malignant cells and TAMs, TECs, and CAFs. Upon activation of specific signaling pathways, *GBA* could enhance malignant behaviors such as proliferation, invasion, metastasis, and angiogenesis. These observations provided preliminary evidence for the oncogenic role of *GBA* in HCC. In addition, we conducted more deep experimental studies subsequently. In HCC patient specimens, we validated the expression pattern of *GBA*, observing a significant upregulation of *GBA* expression in tumor tissues at both mRNA and protein levels. This result was consistent with relevant transcriptomic, proteomic, and IHC data. Next, we found *GBA* was intricately engaged in biological processes, including cell cycle regulation and EMT. Functional experiments and WB analysis further substantiated that *GBA* knockdown notably diminished the proliferative, migratory, and invasive capacity of HCC cells, which aligned with the results of single-cell analysis. Therefore, our findings exhibited novel evidence regarding the role of *GBA* in HCC. *GBA* promoted the malignant behaviors in HCC, including proliferation, invasion, and metastasis. In summary, our findings extended the understanding of PCDI genes in HCC and thereby enhanced the credibility of PCDI as a biomarker.

Subsequently, the PCDI was further validated. The PCDI exhibited robust predictive efficiency for clinical prognosis. In TCGA-LIHC dataset, we observed a significant correlation between the PCDI and clinical staging, pathological grade, T staging, and vascular invasion status among patients. The PCDI also emerged as an independent risk factor for the OS, PFS, DFS, and DSS. The accuracy and stability of the PCDI in predicting prognosis were assessed by ROC curves, C-index curves, and DCA curves, obviously outperforming other clinical indicators. These findings were independently validated in both the GSE76427 and ICGC-LIRI-JP datasets. Moreover, we compared the PCDI with 102 different prognostic models published in recent years. Most models exhibited good performance in the training dataset (TCGA-LIHC). However, the predictive performance obviously declined in the validation datasets (GSE76427 and ICGC-LIRI-JP). This decline should be attributed to overfitting in models developed through a single algorithm, resulting in reduced model generalizability. Notably, despite the decreased predictive performance in the validation datasets, the PCDI maintained superior performance over nearly all other models during the comparative analysis. This suggests that dimension reduction through the combination of machine learning algorithms is an effective approach for improving model generalizability. To assess the practical utility of PCDI in clinical settings, we developed clinical nomograms across the three datasets. Moreover, we observed a significant correlation between PCDI score groups and PCD clusters. Patients in the high PCDI score group and in PCD cluster A demonstrated a substantial overlap in sample distribution, indicating similar unfavorable prognoses and biological functional features. The alignment between PCDI score groups and PCD clusters undeniably bolstered the credibility of PCDI. In conclusion, these findings highlighted the superior predictive performance of the PCDI in clinical prognosis, affirming its suitability as a novel biomarker for prognostic evaluation in HCC patients.

The PCDI exhibited robust predictive efficiency for the immunotherapeutic responses of HCC patients. In the high PCDI score group, we observed a conspicuous immunosuppressive microenvironment characterized by enhanced immunosuppressive cell infiltration, including M2 macrophages, Treg cells, and neutrophils, along with impaired antitumor immune functions such as IFN response and T-cell co-stimulation, resembling the immunological features of cluster A. In addition, we discerned a significant positive correlation between stemness score and PCDI score, aligning with a previous study associating tumor stem cell status with immunological characteristics in solid tumors. This stem cell phenotype was found to inhibit anti-tumor immune functions (66). Given the close relationship between PCDI and immunological characteristics in HCC patients, we further investigated the potential of PCDI for predicting immunotherapeutic responses. Our results indicated that HCC patients in the high PCDI score group displayed elevated gene mutation frequencies. *TP53* was the most frequently mutated gene in the high PCDI score group, while *CTNNB1* was the most frequently mutated one in the low PCDI score group. Studies have shown that *TP53* and *CTNNB1* mutations are common in HCC, usually occurring in the early stages. *TP53* mutations lead to the loss of P53 function and could promote the recruitment of immunosuppressive cells, whereas *CTNNB1* mutations could enhance immune evasion and resistance to immunotherapy in tumor cells (67). Furthermore, we found a significant positive correlation between the PCDI score, TMB and MSI levels, and the expressions of most ICGs. TMB, MSI, and ICG expression patterns were considered crucial indicators for predicting immunotherapeutic responses in tumor patients. It is widely accepted that increased levels of TMB, MSI, and ICG expression correlated with a higher likelihood of positive responses to immunotherapy (33, 68–70). Therefore, we posit that HCC patients with higher PCDI scores could benefit more from immunotherapy. Subsequently, we validated this hypothesis through the TIDE algorithm. By calculating the TIDE scores, we observed that patients in the high PCDI score group exhibited significantly decreased TIDE scores. This suggested that patients with higher PCDI scores were more responsive to immunotherapy. Subsequent correlation analysis validated that the immunotherapeutic response rates of patients in the high PCDI score group were significantly higher than those in the low PCDI score group. These results provided more compelling evidence that the PCDI could predict immunotherapy responses in HCC patients. Thereafter, a more comprehensive study was conducted to assess the predictive ability of PCDI in immunotherapeutic responses across multiple immunotherapy cohorts. Our findings revealed that in the IMvigor210, GSE176307, Checkmate, GSE179351, GSE103668, and GSE78220 cohorts, patients who responded to immunotherapy were predominantly found in the high PCDI score group. In the GSE35640 and GSE120644 cohorts, patients who responded to immunotherapy were primarily in the low PCDI score group. In the GSE91061 cohort, immunotherapeutic responses seemed unrelated to PCDI scores. In summary, the PCDI demonstrated excellent predictive capability regarding immunotherapy responses. Higher PCDI scores were associated with a greater likelihood of tumor patients benefiting from immunotherapy. These results highlighted the PCDI as a valuable tool for predicting the immunotherapy responses of tumor patients. In particular, HCC patients with higher PCDI scores were more suitable candidates for immunotherapy.

Additionally, we found that the PCDI could be employed to predict the chemotherapeutic sensitivity of patients with HCC. The expression levels of most CRRGs in patients with HCC showed a significant positive correlation with the PCDI score. This observation suggested that the PCDI could serve as an effective indicator for assessing chemotherapeutic resistance. Patients with higher PCDI scores may exhibit heightened resistance to chemotherapy. In the two HCC treatment cohorts, GSE109211 and GSE104580, we observed that patients with lower PCDI scores were more responsive to sorafenib and TACE treatments. When comparing the imputed sensitivity scores of drugs, HCC patients with lower PCDI scores demonstrated heightened sensitivity to oxaliplatin, whereas those with higher PCDI scores exhibited heightened sensitivity to inhibitors of cell mitosis and proliferation, such as paclitaxel, docetaxel, and vinblastine, as well as certain targeted drugs and small molecule inhibitors such as cediranib, bortezomib, MIM1, MK-1775, and WIKI4. In summary, the PCDI exhibited remarkable predictive efficacy in assessing the responses of HCC patients to various therapies, including immunotherapy. Overall, it holds promise as a novel biomarker for guiding personalized treatment in HCC.

Although we have demonstrated the robust performance and clinical value of the PCDI, it is necessary to recognize several constraints inherent in this study. Firstly, the data used here were all sourced from public databases, classifying it as a retrospective study. During the data processing phase, we excluded samples with incomplete clinical data, which reduced the usage of samples and might have influenced the analytical outcomes. Consequently, large-scale prospective studies are still necessary to comprehensively evaluate the precise value of the PCDI. Secondly, we provided a comprehensive landscape of the PCDI genes across multiple levels, including the transcriptome, proteome, and single-cell analyses. We also discussed the role of PCDI genes in HCC development based on existing research. Moreover, we contributed new experimental evidence supporting the role of GBA in the progression of HCC. All of these enhance the reliability of the PCDI as a biomarker for HCC. However, further research is necessary to elucidate the detailed mechanisms by which these genes regulate HCC progression and therapy responses. Lastly, additional therapeutic cohorts involving HCC patients are needed to further validate the predictive value of the PCDI in treatment responses among HCC patients.

In conclusion, we systematically analyzed the correlation between 14 programmed cell death modes and the clinical characteristics and biological patterns of HCC. We constructed a precise and robust PCDI model through a comprehensive array of machine-learning algorithms. The PCDI demonstrated remarkable accuracy in predicting the prognosis and treatment responses of HCC patients. It served as an effective biomarker for heterogeneity delineation and risk stratification. The application of PCDI has the potential to facilitate personalized treatment and clinical management for HCC patients, representing a significant contribution to clinical practice.

## Data availability statement

The datasets presented in this study can be found in online repositories. The names of the repository/repositories and accession number(s) can be found in the article/**Supplementary Material**.

## Ethics statement

The studies involving humans were approved by Tongji Hospital Research Ethics Committee. The studies were conducted in accordance with the local legislation and institutional requirements. The participants provided their written informed consent to participate in this study.

## Author contributions

YS: Conceptualization, Data curation, Formal analysis, Investigation, Methodology, Visualization, Writing – original draft. YF: Investigation, Visualization, Writing – original draft. PO: Data curation, Writing – review & editing. KZ: Data curation, Writing – review & editing. XL: Data curation, Writing – review & editing. ZD: Project administration, Supervision, Writing – review & editing. JW: Funding acquisition, Project administration, Resources, Supervision, Writing – review & editing.
