# Supplementary material for: Identifying the programmed cell death index of hepatocellular carcinoma for prognosis and therapy response improvement by machine learning: a bioinformatics analysis and experimental validation
Source: Front Immunol. 2023 Dec 19;14:1298290. doi: 10.3389/fimmu.2023.1298290 (PMC10759150; doi:10.3389/fimmu.2023.1298290)
Supplement: Supplementary file 1 [file DataSheet_1.docx]

**Supplementary Figures**

**
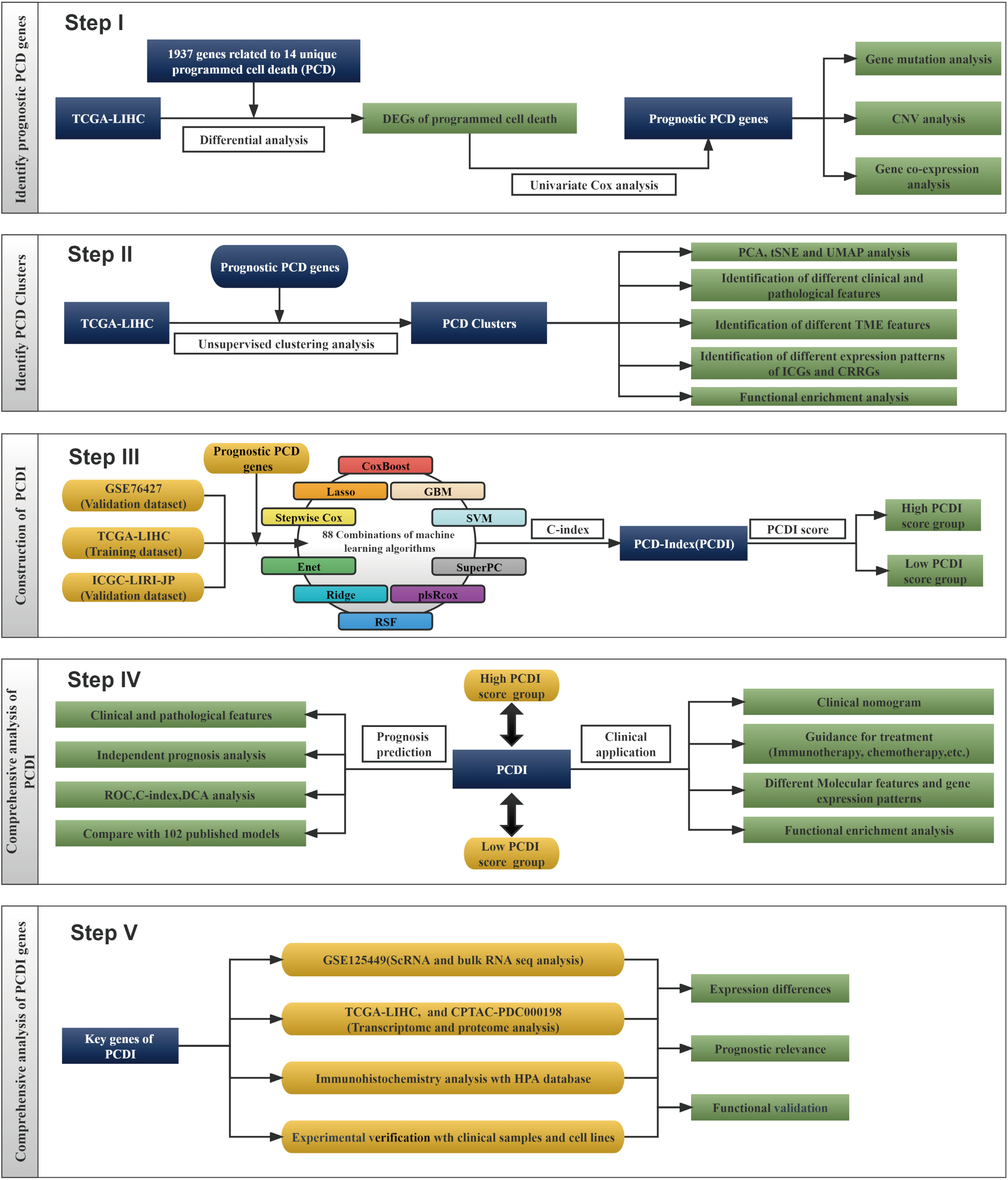
**

**Figure S1. The overall design of this study visualized as the flowchart.**

**
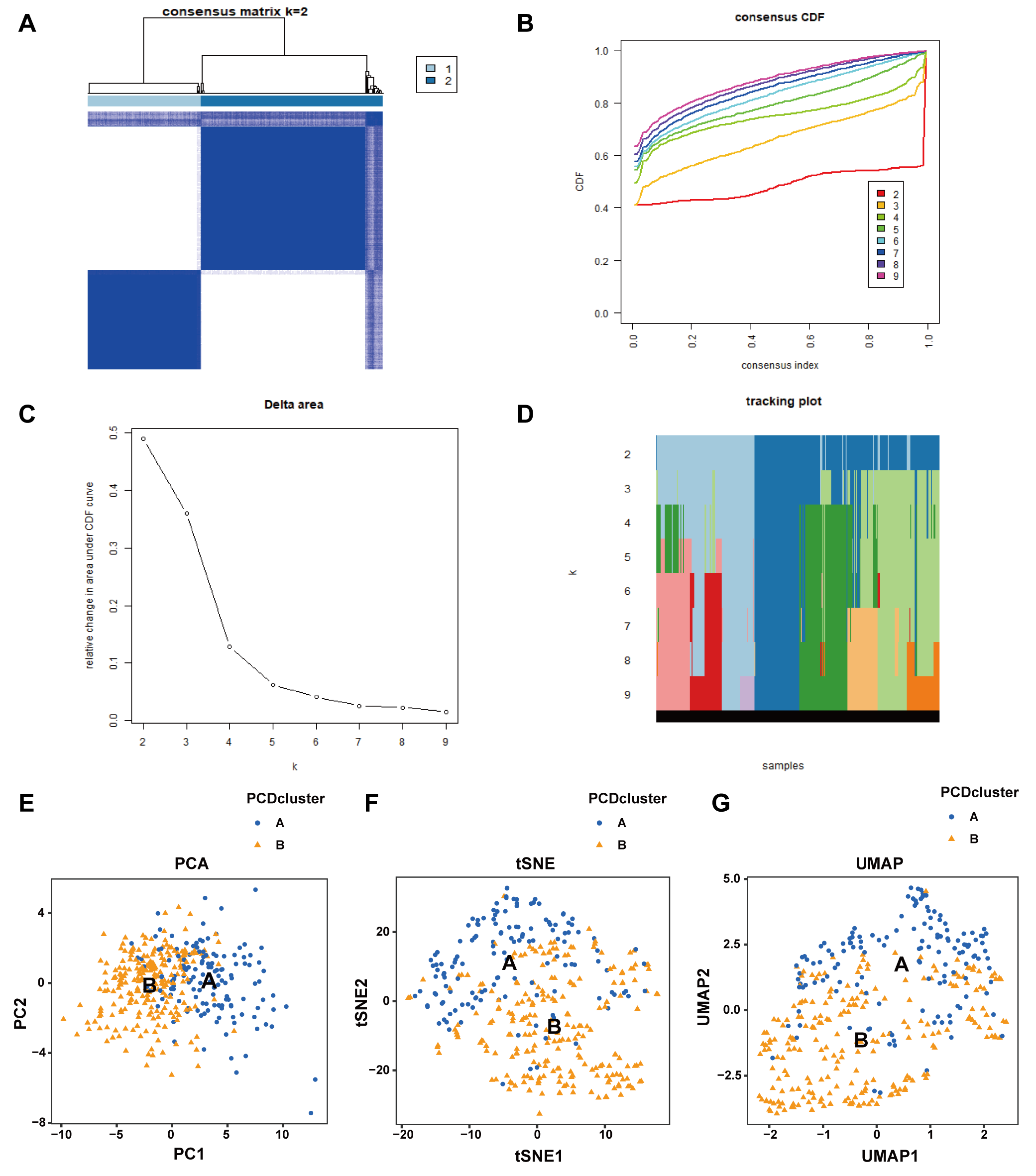
Figure S2. Identification of PCD clusters in the TCGA-LIHC dataset. (A)** HCC patients were divided into two clusters (K=2) according to PCD-related gene expression levels by unsupervised clustering. **(B)** Consensus distributions for k from 2 to 9 visualized by CDF curve plot. **(C)** Areas under CDF curves for k from 2 to 9 visualized by Delta Area plot. **(D)** Distributions of HCC patients for k from 2 to 9 visualized by tracking plot. **(E)** Distributions of HCC patients between the two clusters visualized by PCA plot. **(F)** Distributions of HCC patients between the two clusters visualized by t-SNE plot. **(G)** Distributions of HCC patients between two clusters visualized by UMAP plot.

**
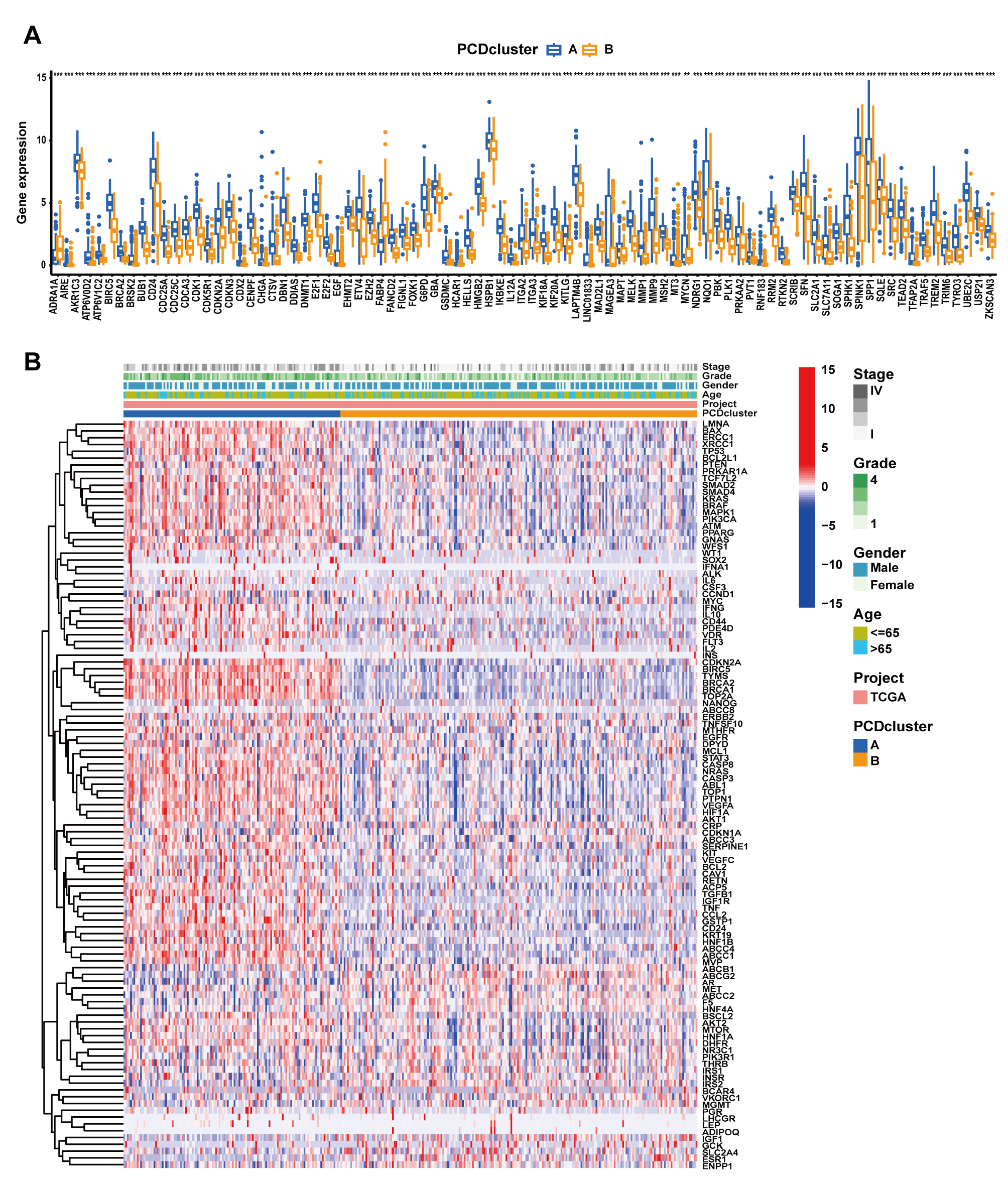
Figure S3. The correlation between the PCD clusters, clinicopathological characteristics, and expression patterns of PCD-related genes and CRRGs. (A)** Different PCD-related gene expression patterns between the PCD clusters. **(B)** Different clinicopathological characteristics and CRRG expression patterns between the PCD clusters.

**
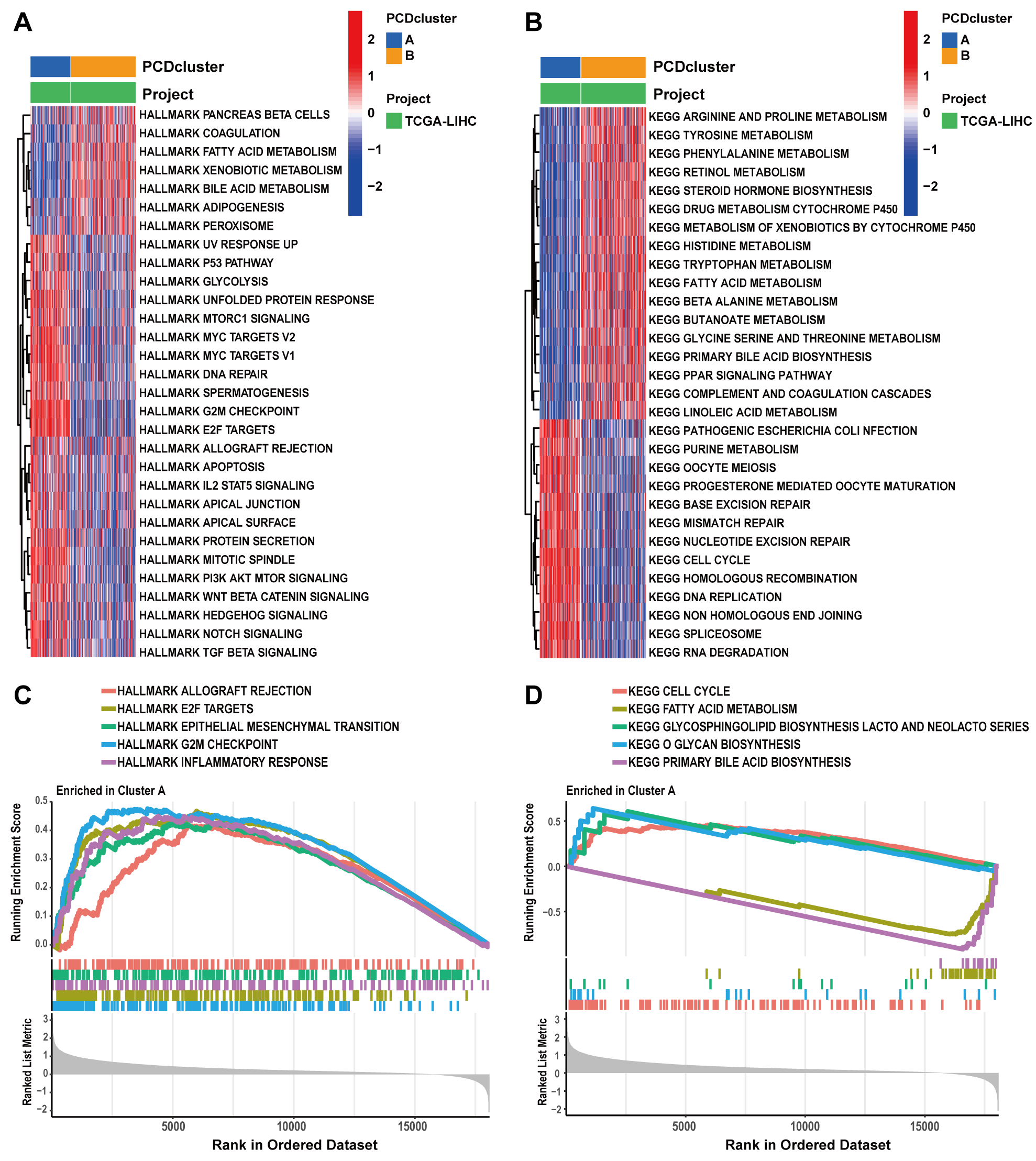
Figure S4. GSVA and GSEA analysis between the PCD clusters. (A-B)** GSVA analysis with HALLMARK and KEGG terms between the two PCD clusters. **(C-D)** GSEA analysis with HALLMARK and KEGG terms between the two PCD clusters.

**
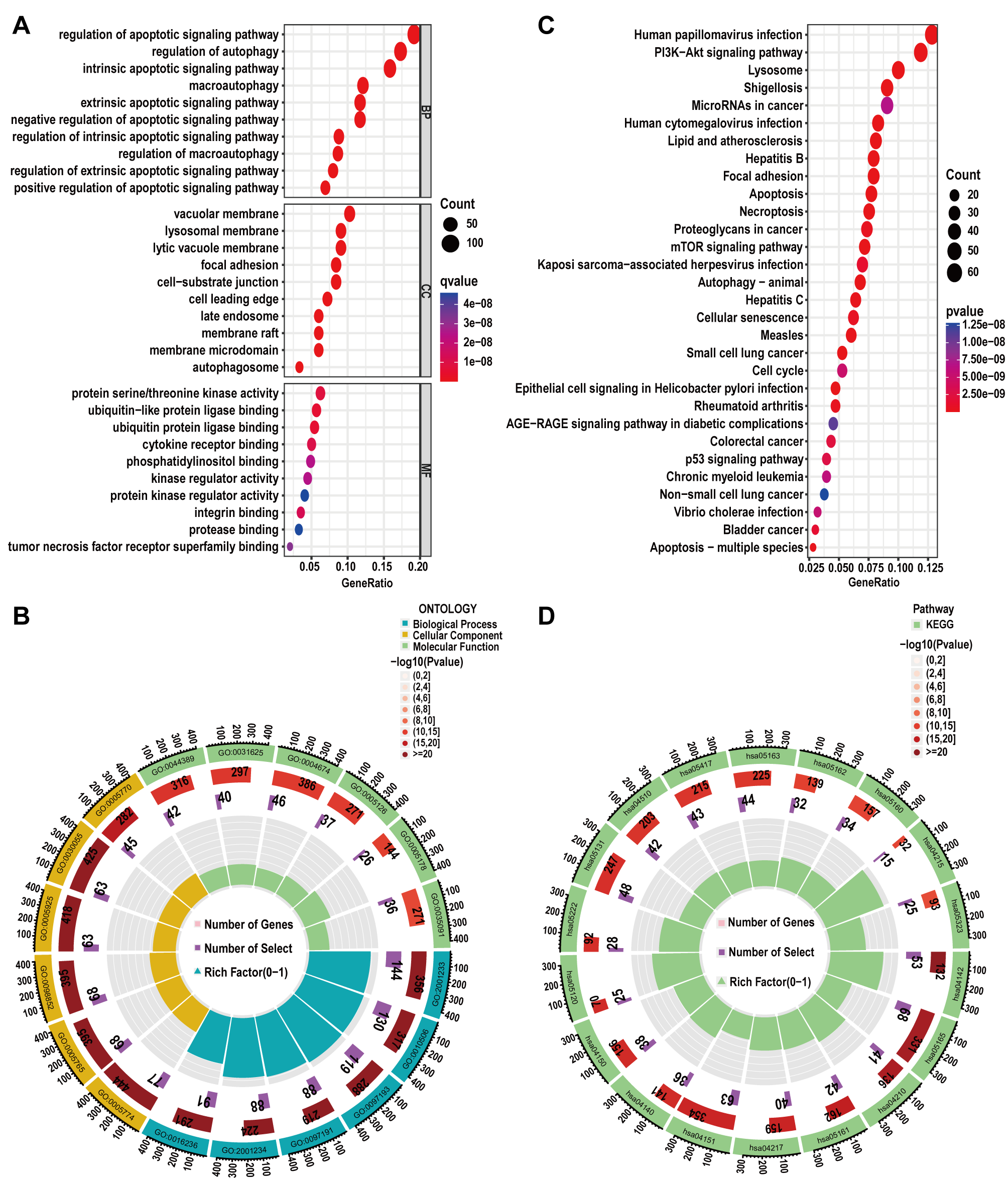
Figure S5. GO/KEGG functional enrichment analysis between two PCD clusters. (A-B)** GO functional enrichment analysis between the two PCD clusters. **(C-D)** KEGG functional enrichment analysis between the two PCD clusters.

**
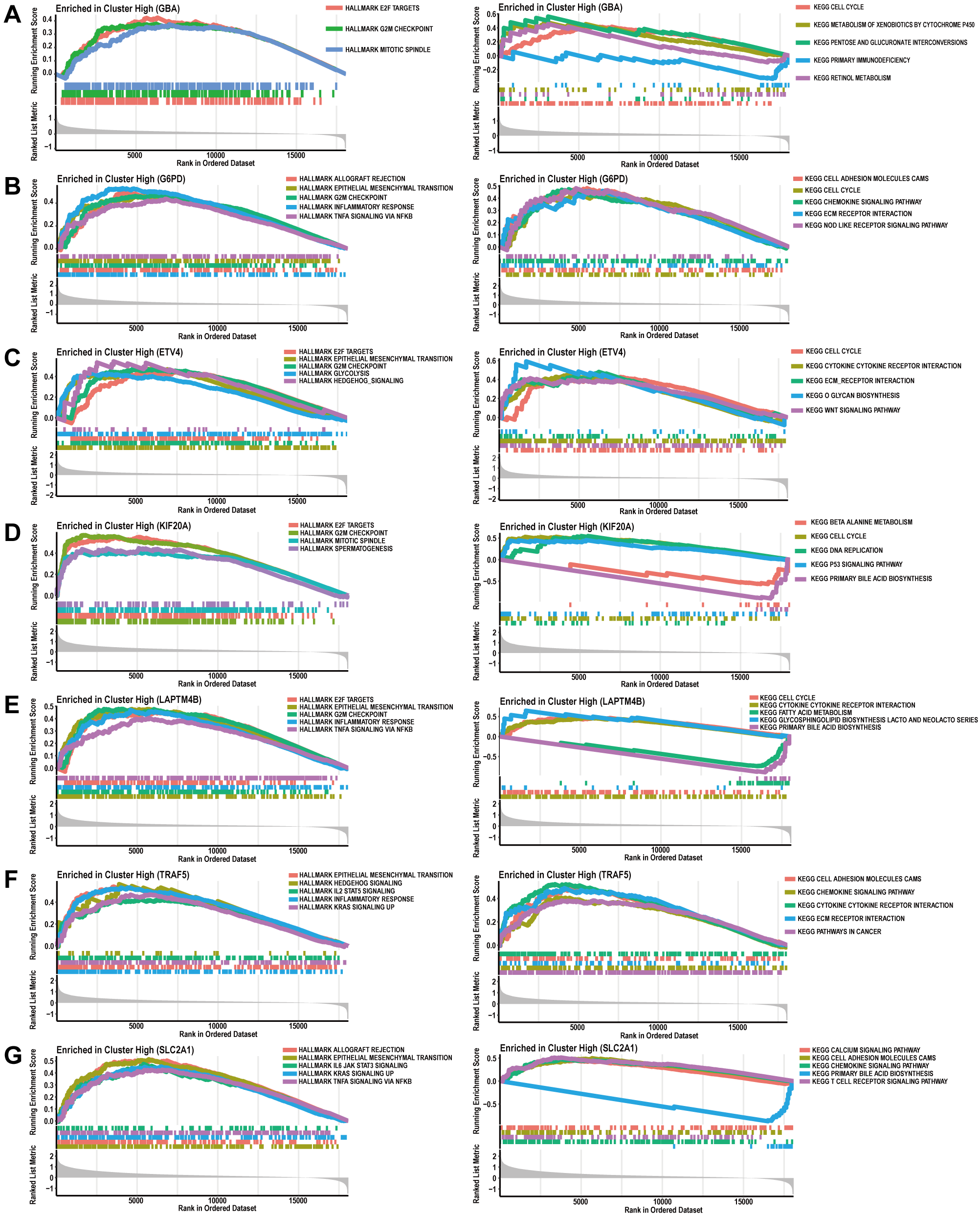
Figure S6. Biological function analysis for PCDI genes.** GSEA analysis with HALLMARK and KEGG terms for **(A)** GBA **(B)** G6PD **(C)** ETV4 **(D)** KIF20A **(E)** LAPTM4B **(F)** TRAF5 **(G)** SLC2A1

**
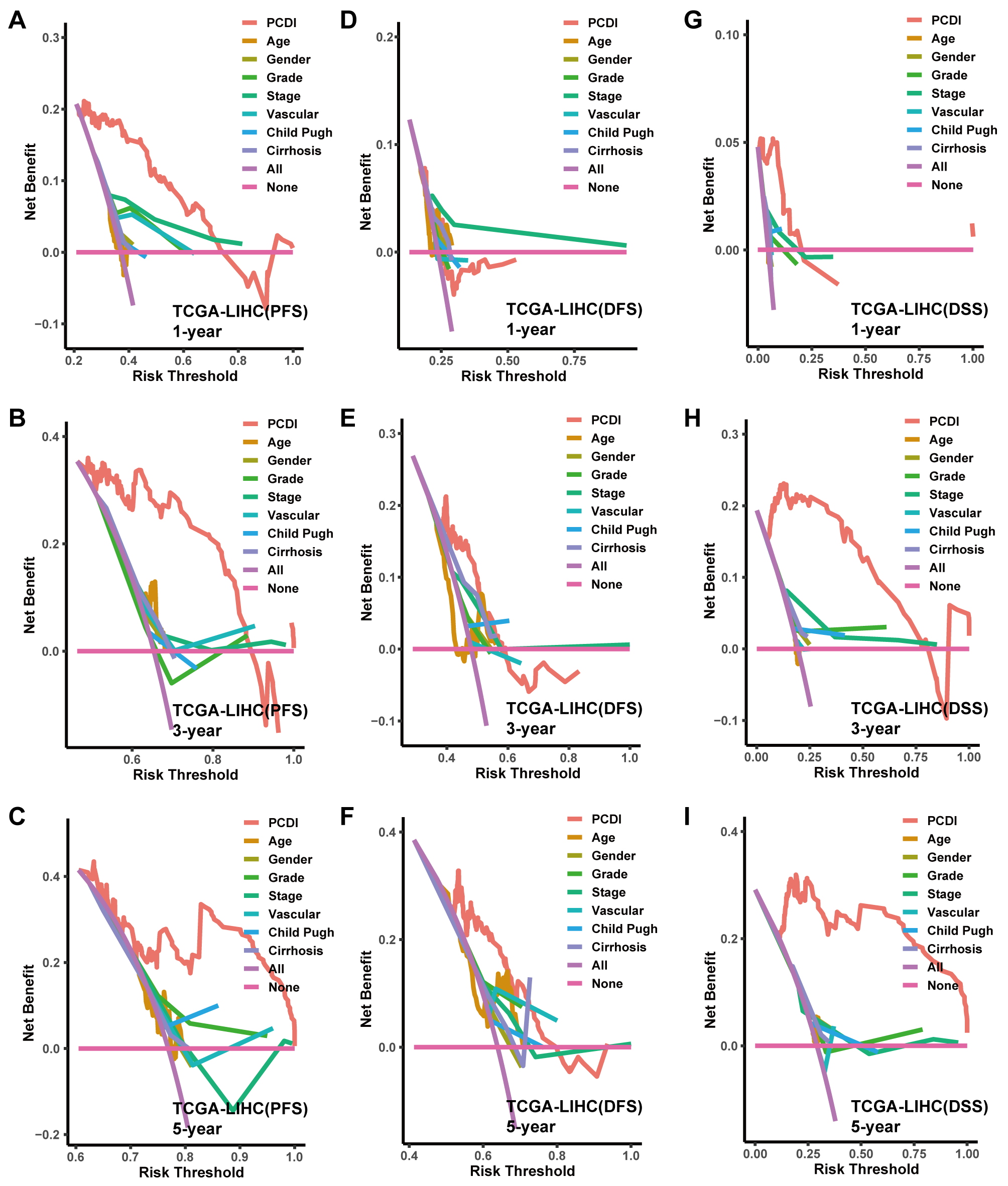
Figure S7. Comparing the prognostic predictive efficacy of PCDI and clinicopathological characteristics based on other survival indicators. (A-C)** DCA curves of PCDI and clinicopathological characteristics for predicting PFS in the TCGA-LIHC dataset. **(D-F)** DCA curves of PCDI and clinicopathological characteristics for predicting DFS in the TCGA-LIHC dataset. **(G-I)** DCA curves of PCDI and clinicopathological characteristics for predicting DSS in the TCGA-LIHC dataset.

**
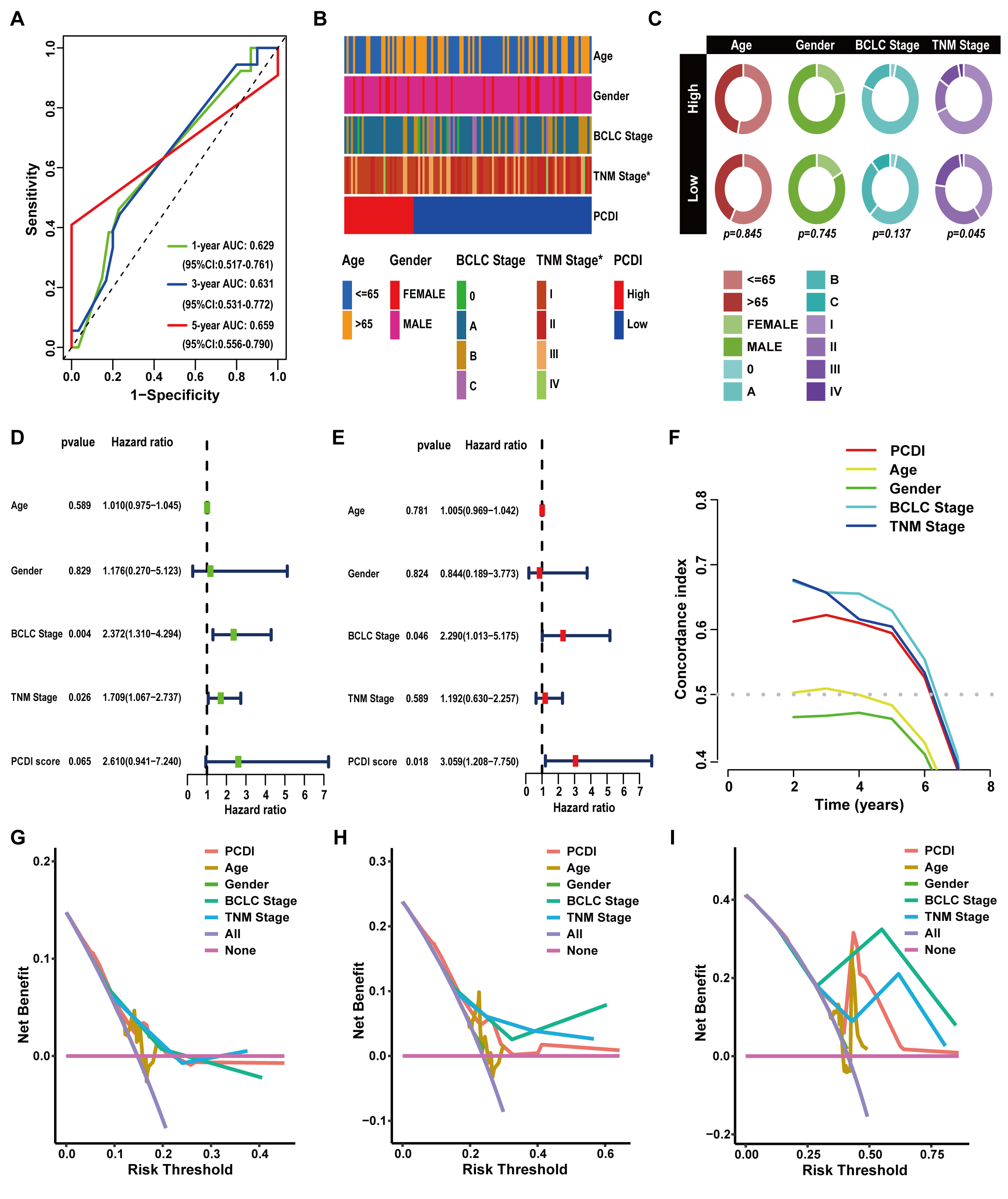
Figure S8. Further validation of the prognostic predictive value of PCDI in the GSE76427 dataset. (A)** Validation of the predictive accuracy of PCDI for OS with ROC curves. **(B-C)** Correlation analysis of clinicopathological characteristics and PCDI. **(D)** Univariate Cox analysis revealing the impacts of PCDI and clinicopathological characteristics on OS. **(E)** Multivariate Cox analysis revealing the impacts of PCDI and clinicopathological characteristics on OS. **(F)** Comparing the prognostic predictive efficacy of PCDI and clinicopathological characteristics for OS with C-index curves. **(G-I)** Comparing the prognostic predictive efficacy of PCDI and clinicopathological characteristics for OS with DCA curves.

**
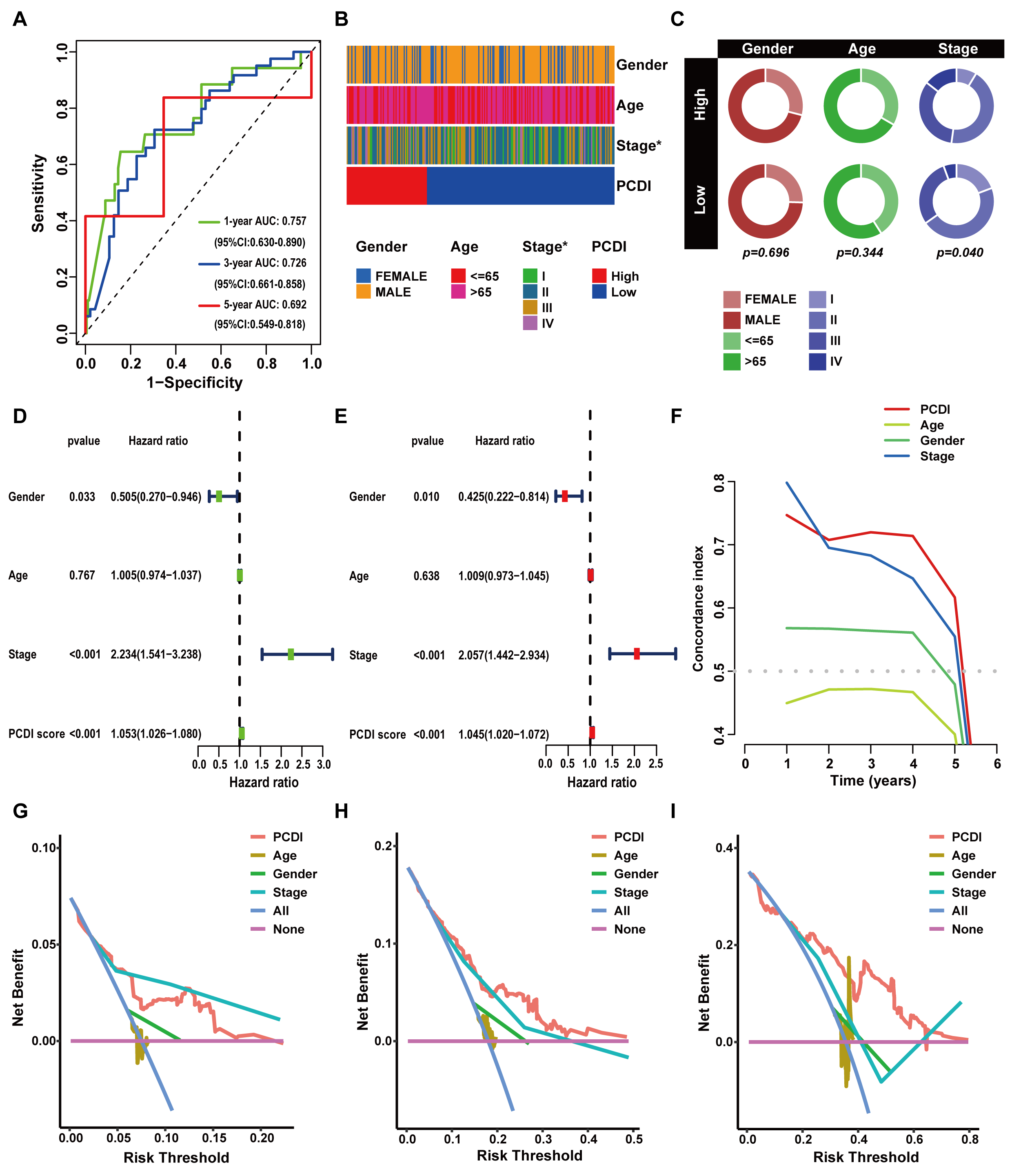
Figure S9. Further validation of the prognostic predictive value of PCDI in the ICGC-LIRI-JP dataset. (A)** Validation of the predictive accuracy of PCDI for OS with ROC curves. **(B-C)** Correlation analysis of clinicopathological characteristics and PCDI. **(D)** Univariate Cox analysis revealing the impacts of PCDI and clinicopathological characteristics on OS. **(E)** Multivariate Cox analysis revealing the impacts of PCDI and clinicopathological characteristics on OS. **(F)** Comparing the prognostic predictive efficacy of PCDI and clinicopathological characteristics for OS with C-index curves. **(G-I)** Comparing the prognostic predictive efficacy of PCDI and clinicopathological characteristics for OS with DCA curves.


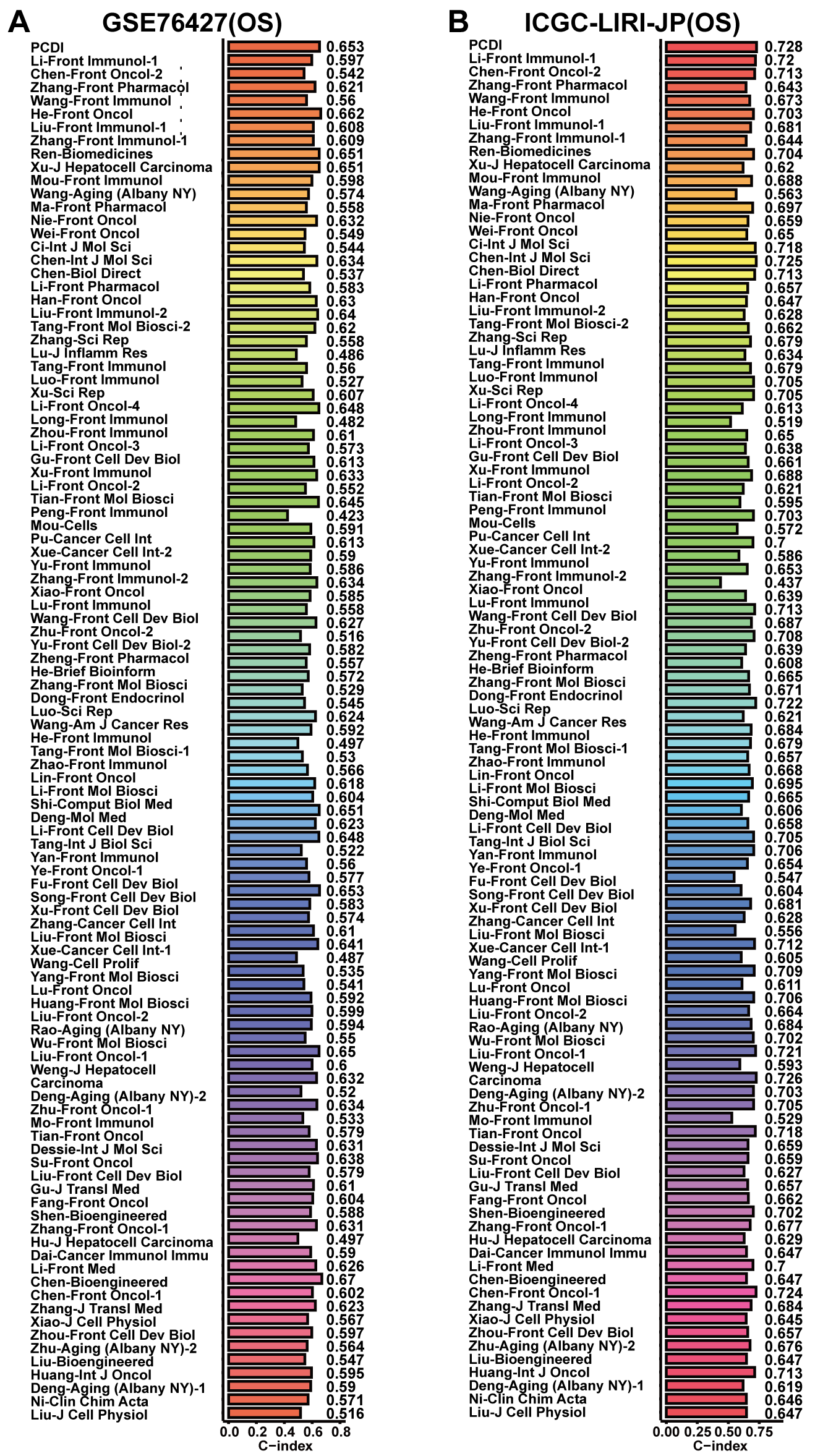


**Figure S10. Comparison of the predictive value between PCDI and other models in GSE76427 and ICGC-LIRI-JP datasets. (A)** Comparing the prognostic predictive efficacy of PCDI and other published models for OS in the GSE76427 dataset by C-index analysis. **(B)** Comparing the prognostic predictive efficacy of PCDI and other published models for OS in the ICGC-LIRI-JP dataset by C-index analysis.

**
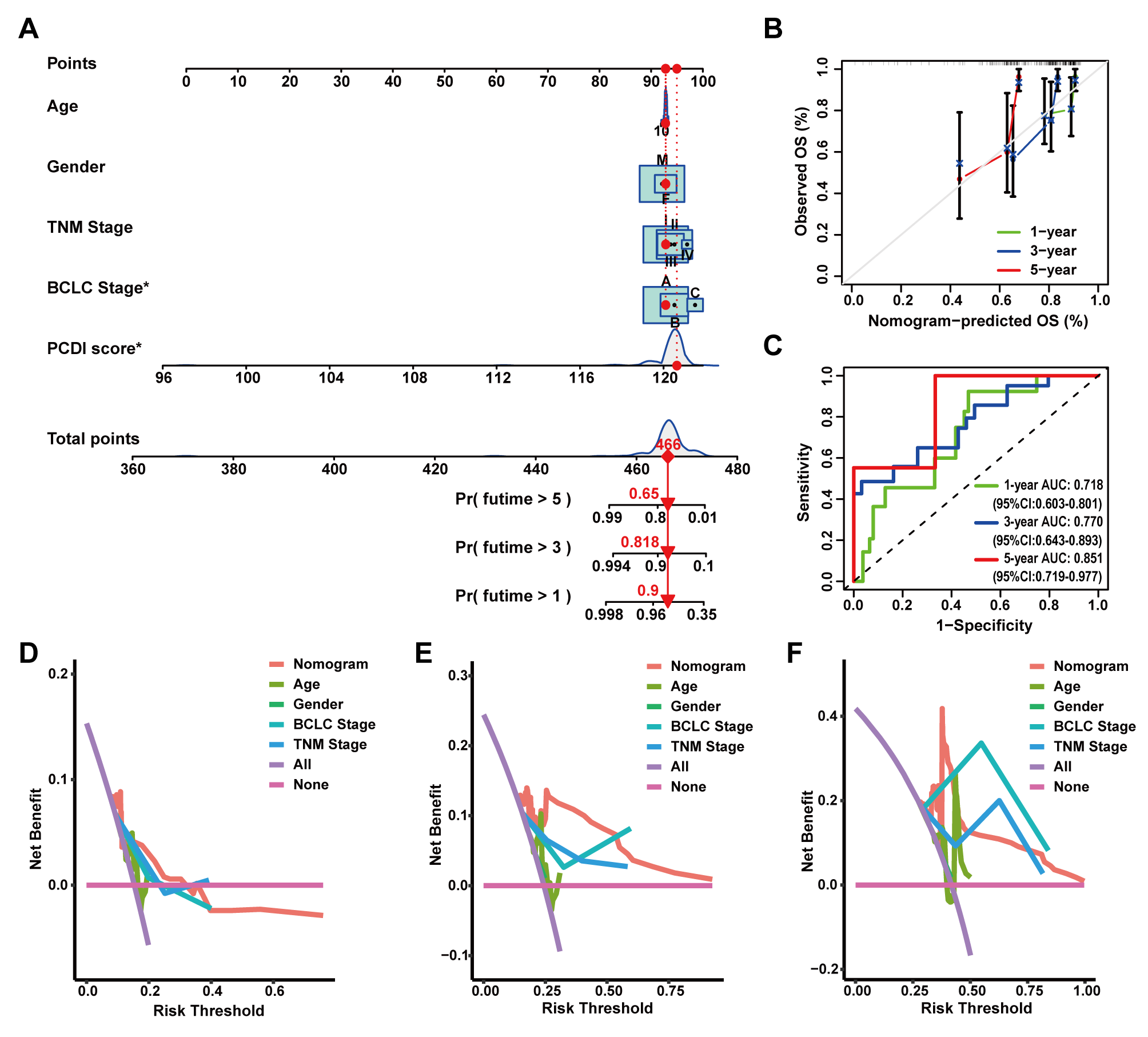
Figure S11. Further validation of nomogram based on PCDI and clinicopathological characteristics in the GSE76427 dataset. (A)** Construction of a nomogram with PCDI and clinicopathological characteristics for predicting OS. **(B)** Evaluating the predictive accuracy of nomogram for OS with calibration curves. **(C)** Evaluating the predictive accuracy of nomogram for OS with ROC curves. **(D-F)** Comparing the predictive efficacy of nomogram and clinicopathological characteristics for OS with DCA curves.

**
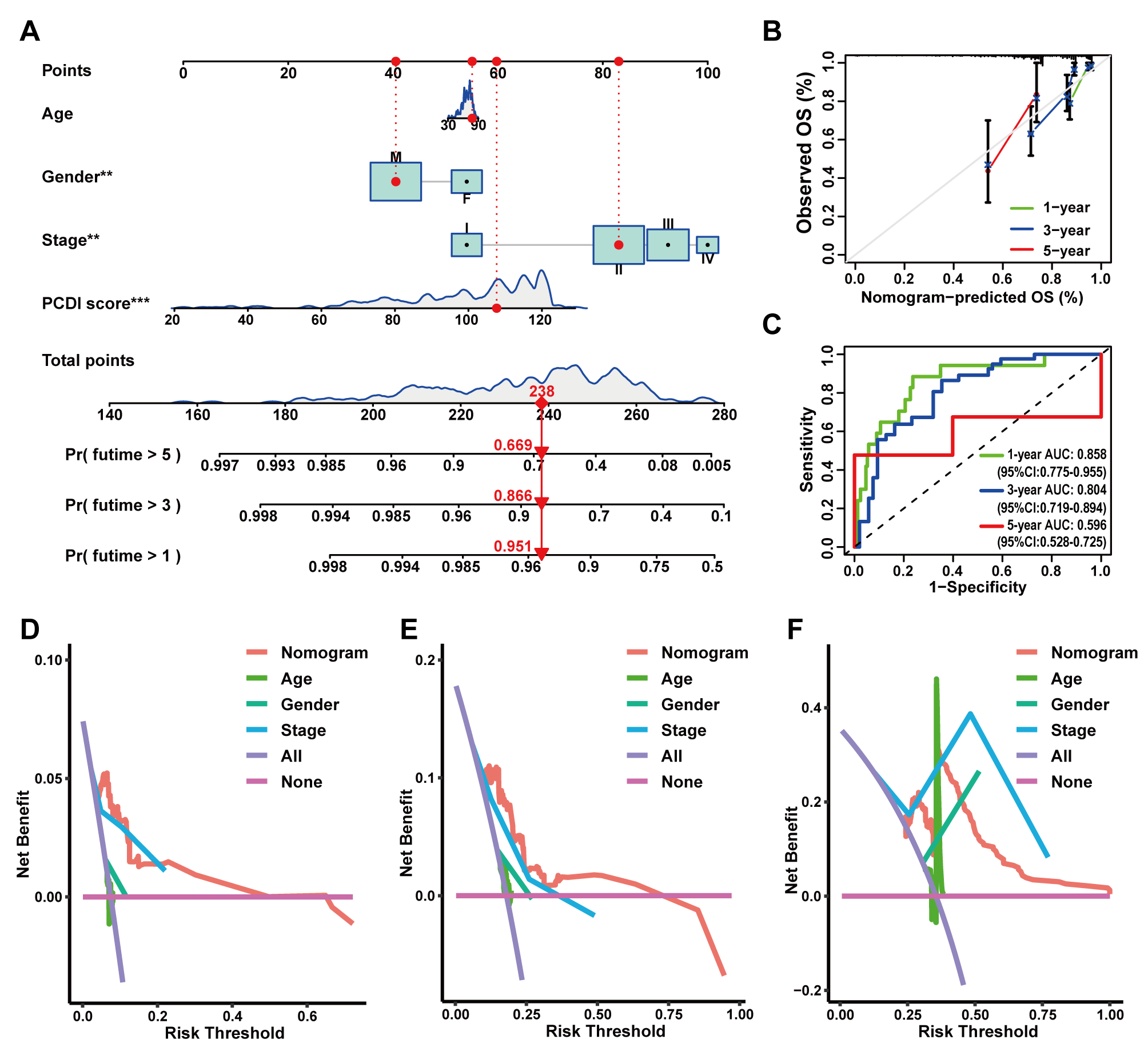
Figure S12. Further validation of nomogram based on PCDI and clinicopathological characteristics in ICGC-LIRI-JP dataset. (A)** Construction of a nomogram with PCDI and clinicopathological characteristics for predicting OS. **(B)** Evaluating the predictive accuracy of nomogram for OS with calibration curves. **(C)** Evaluating the predictive accuracy of nomogram for OS with ROC curves. **(D-F)** Comparing the predictive efficacy of nomogram and clinicopathological characteristics for OS with DCA curves.

**
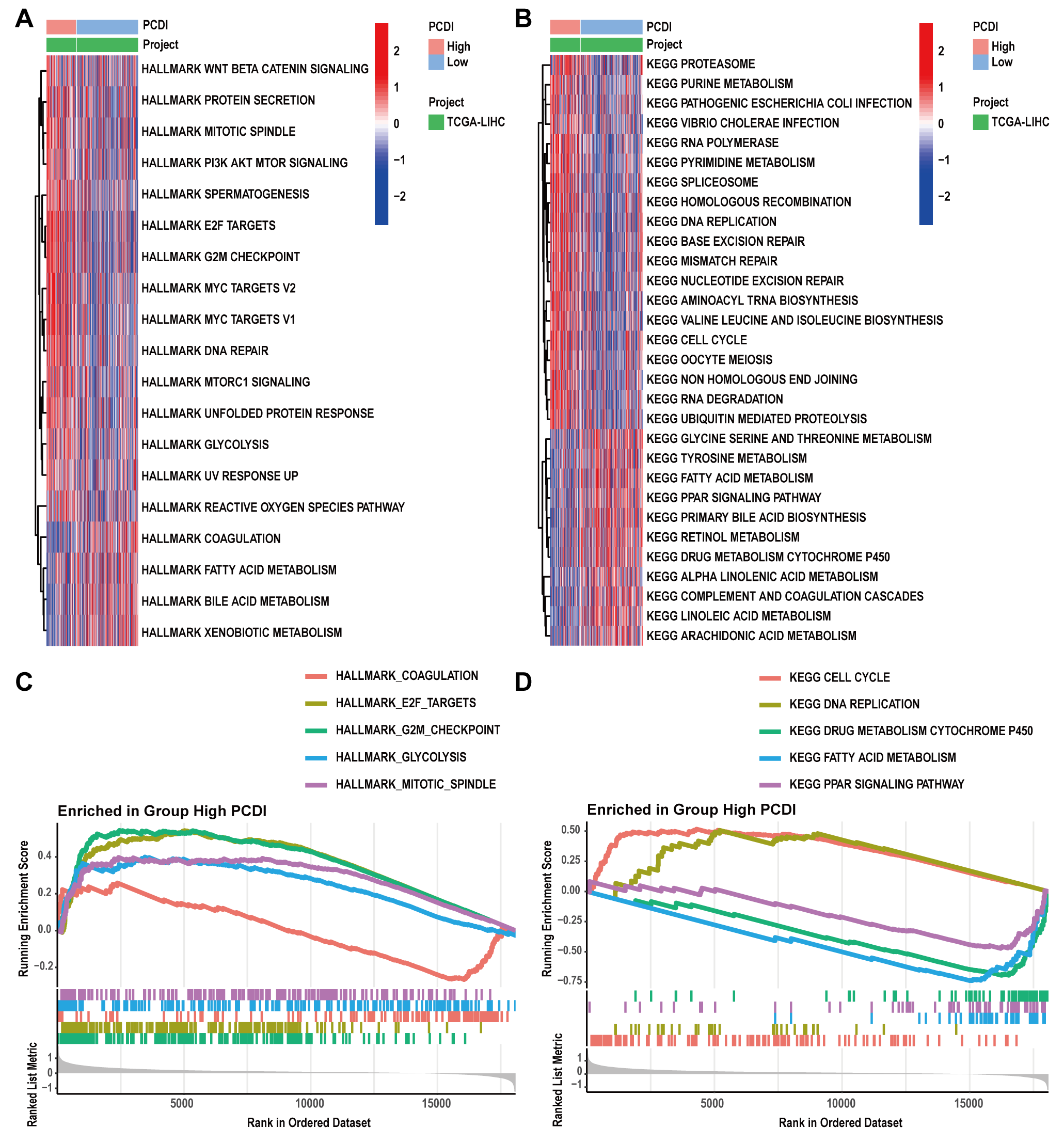
Figure S13. GSVA and GSEA analysis between PCDI score groups. (A-B)** GSVA analysis with HALLMARK and KEGG terms between the high and low PCDI score groups. **(C-D)** GSEA analysis with HALLMARK and KEGG terms between the high and low PCDI score groups.

**
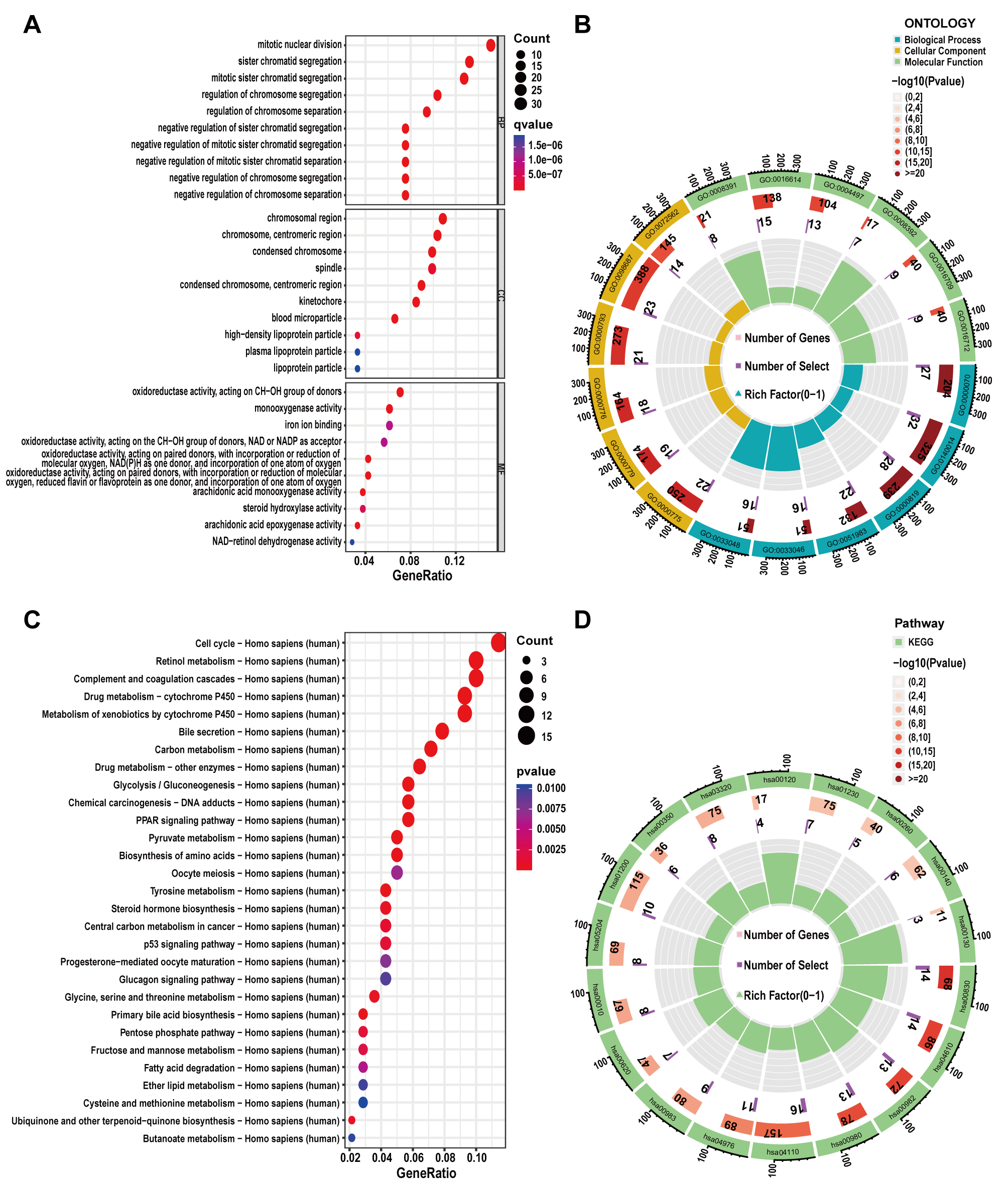
Figure S14. GO/KEGG functional enrichment analysis between PCDI score groups. (A-B)** GO functional enrichment analysis between high and low PCDI score groups. **(C-D)** KEGG functional enrichment analysis between high and low PCDI score groups.

**
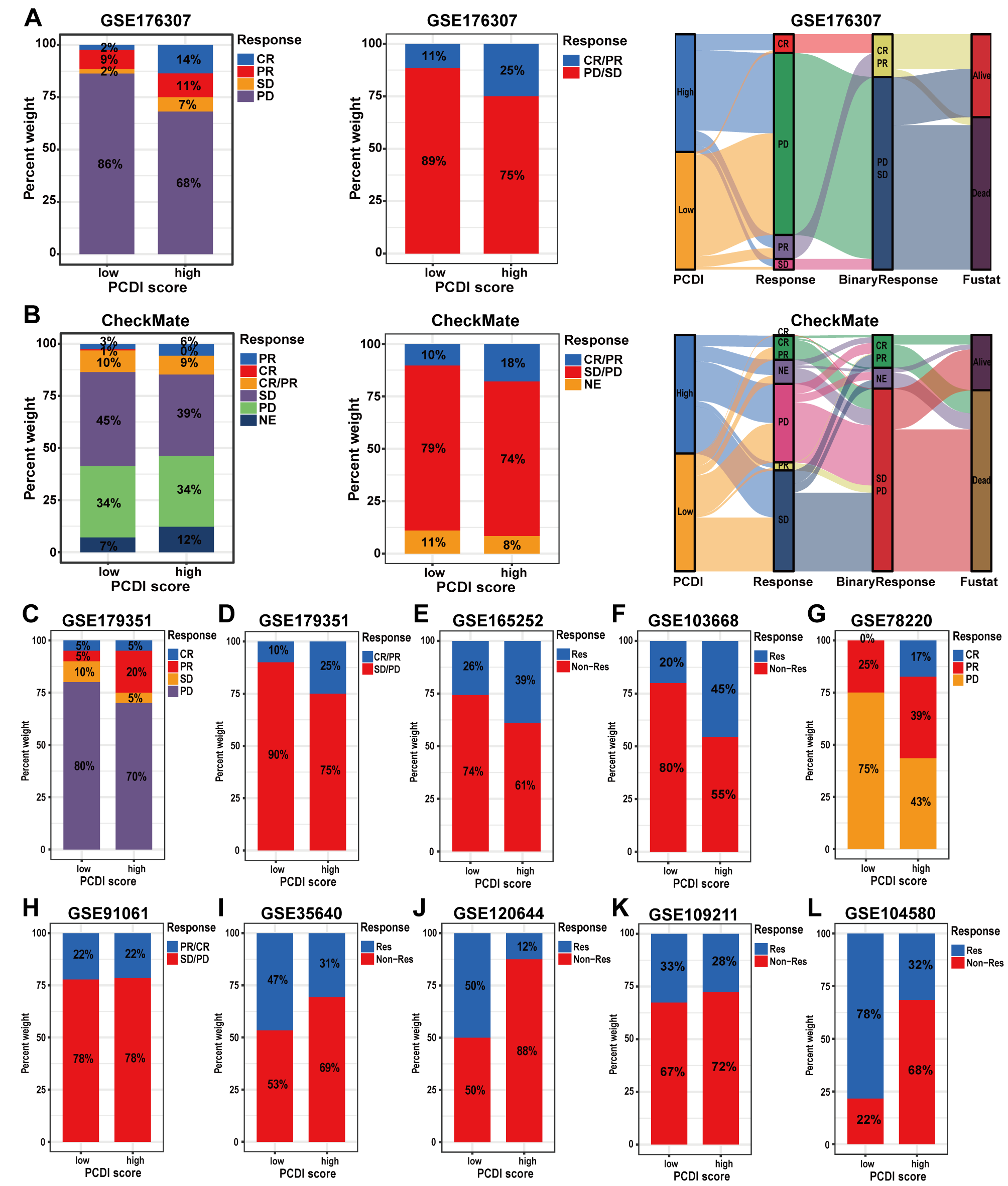
Figure S15. Further validation for the predictive value of PCDI in treatment response within more cohorts. (A-J)** Distribution of patients with different immunotherapy response statuses across PCDI score groups. **(K)** Distribution of patients with different sorafenib therapy response statuses across PCDI score groups. **(L)** Distribution of patients with different TACE therapy response statuses across PCDI score groups.

**
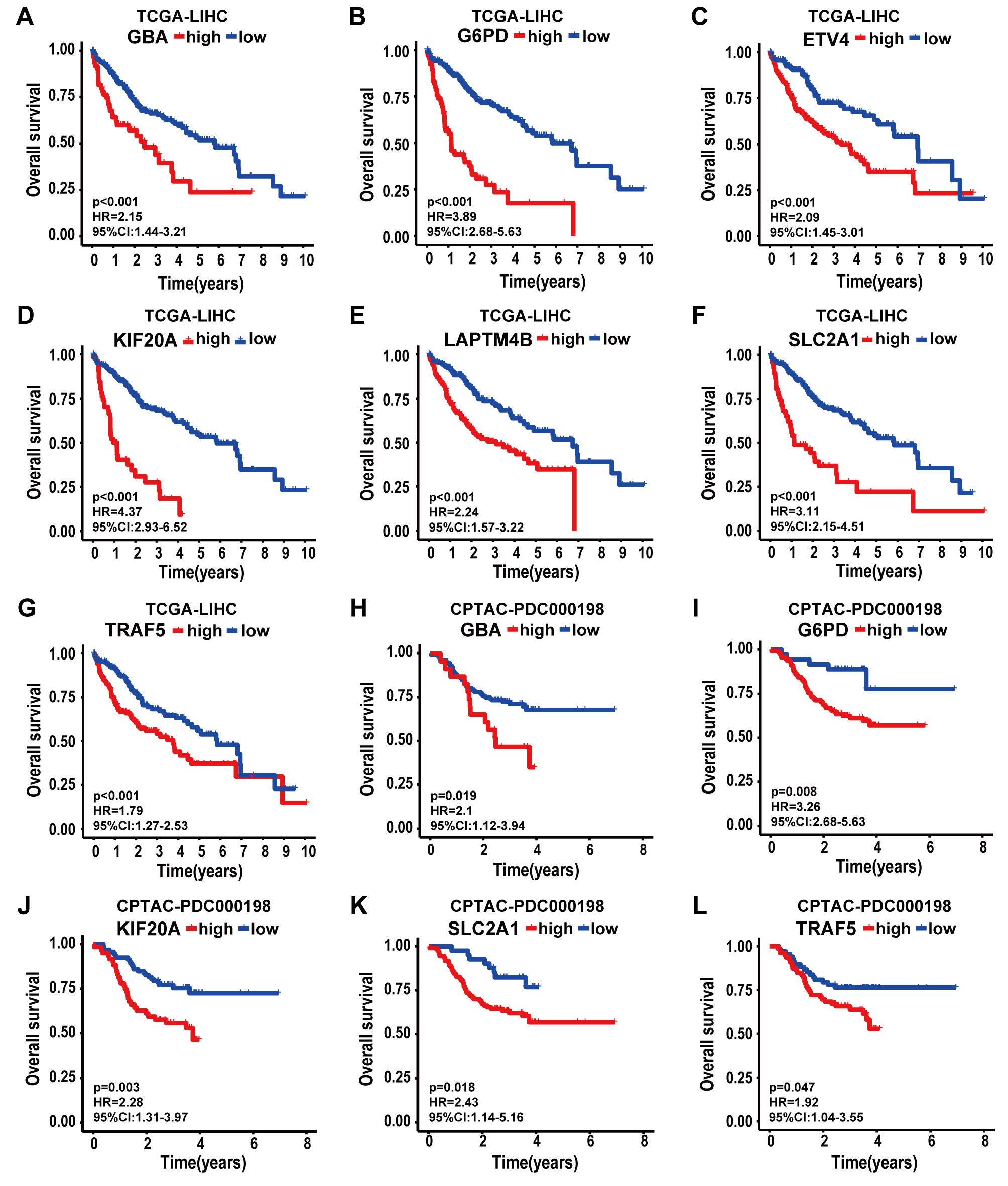
Figure S16. Different OS statuses between patients with high and low PCDI gene expression. (A-G)** Survival analysis of patients with high and low PCDI gene expression (mRNA level) in the TCGA-LIHC dataset. **(H-L)** Survival analysis of patients with high and low PCDI gene expression (protein level) in the CPTAC-PDC000198 dataset.
